# Identification and preliminary analysis of hub genes associated with bladder cancer progression by comprehensive bioinformatics analysis

**DOI:** 10.1038/s41598-024-53265-z

**Published:** 2024-02-02

**Authors:** Han Wang, Junjie Liu, Yanyan Lou, Yang Liu, Jieqing Chen, Xinhui Liao, Xiuming Zhang, Chengzhi Zhou, Hongbing Mei, Aifa Tang

**Affiliations:** 1 Department of Urology, The First Affiliated Hospital of Shenzhen University, Shenzhen Second People’s Hospital, Shenzhen, China; 2 Guangdong Key Laboratory of Systems Biology and Synthetic Biology for Urogenital Tumors, The First Affiliated Hospital of Shenzhen University, Shenzhen Second People’s Hospital, Shenzhen, China; 3 Medical Laboratory of Shenzhen Luohu People’s Hospital, The Third Affiliated Hospital of Shenzhen University, Shenzhen, China; 4 Department of Oncology, Yantian District People’s Hospital, Shenzhen, China; 5 Pulmonary and Critical Care Medicine, Guangzhou Institute of Respiratory Health, National Clinical Research Center for Respiratory Disease, National Center for Respiratory Medicine, State Key Laboratory of Respiratory Diseases, The First Affiliated Hospital of Guangzhou Medical University, Guangzhou, China; 6 Science and Educational Center of Shenzhen Luohu People’s Hospital, The Third Affiliated Hospital of Shenzhen University, Shenzhen, Guangdong China

**Keywords:** Cancer, Diseases, Oncology

## Abstract

Bladder cancer (BC) is a crisis to human health. It is necessary to understand the molecular mechanisms of the development and progression of BC to determine treatment options. Publicly available expression data were obtained from TCGA and GEO databases to spot differentially expressed genes (DEGs) between cancer and normal bladder tissues. Weighted co-expression networks were constructed, and Gene Ontology (GO), Kyoto Encyclopedia of Genes and Genomes (KEGG) pathway enrichment analyses were performed. Associations in hub genes, immune infiltration, and immune therapy were evaluated separately. Protein–protein interaction (PPI) networks for the genes identified in the normal and tumor groups were launched. 3461 DEGs in the TCGA dataset and 1069 DEGs in the GSE dataset were identified, including 87 overlapping genes between cancer and normal bladder groups. Hub genes in the tumor group were mainly enriched for cell proliferation, while hub genes in the normal group were related to the synthesis and secretion of neurotransmitters. Based on survival analysis*, CDH19, RELN, PLP1,* and *TRIB3* were considerably associated with prognosis (P < 0.05). *CDH19, RELN, PLP1,* and *TRIB3* may play important roles in the development of BC and are potential biomarkers in therapy and prognosis.

## Introduction

Bladder cancer (BC) is a malignant tumour with the fourth highest incidence among all tumours and the highest incidence among urinary system cancers in men. According to recent cancer statistics analyses, approximately 81,400 newly diagnosed BC cases and 17,980 deaths occur annually^1^. BC can be divided into superficial and highly differentiated diseases, and patients show a good prognosis and quality of life in superficial cancer, while high mortality in poorly differentiated cancer. Exploring the mechanism of the occurrence and development of bladder cancer is important to maintain the long-term survival rate of patients^2^. The superficial phenotype of BC generally corresponds to non-muscle-invasive BC (NMIBC), which accounts for 70–80% of new BC cases and is usually cured by transurethral resection of the bladder tumour. Still one of the major challenges in the treatment of NMIBC is the high recurrence rate, which is up to 70%. Moreover, about 30% of recurrent bladder cancers will be converted into MIBC, requiring lifelong follow-up monitoring. The highly lethal type is generally classified as muscle-invasive BC (MIBC) in clinical terms, which is routinely treated by partial cystectomy and radical cystectomy. However, owing to distant metastasis in the early stage, many patients cannot undergo surgery, which leads to a poor prognosis for patients with MIBC^3–5^. Therefore, the clinical treatment of BC usually involves a combination of surgery, chemotherapy, radiotherapy, and biotherapy. Nevertheless, progression from NMIBC towards MIBC cannot be effectively inhibited. Targets for early diagnosis and treatment of MIBC are needed urgently.

Genomic and chip data are becoming increasingly abundant owing to advance in sequencing methods. Numerous algorithms have been developed to screen for diagnostic and therapeutic targets based on expression data. In particular, weighted gene co-expression network analysis (WGCNA), a system biology algorithm aimed at: (1) finding gene co-expression modules; (2) exploring relationships between gene networks and clinical phenotypes^6,7^; (3) differential gene expression analyses were conducted to discern expression disparities between tumor and normal tissues; (4) clarifying molecular mechanisms underlying the regulation of biological processes^8^.WGCNA can be used to effectively screen for potential targets for the early diagnosis and treatment of BC.

In this study, differential gene co-expression was identified through mRNA expression profiles in TCGA and GEO databases analyses, four interested genes were verified in multiple databases and several experiments, including qRT-PCR, western blot and immunohistochemical. Our study paved a potential road for the clinical diagnosis and four treatment targets for BC.

## Materials and methods

**Figure 1:**
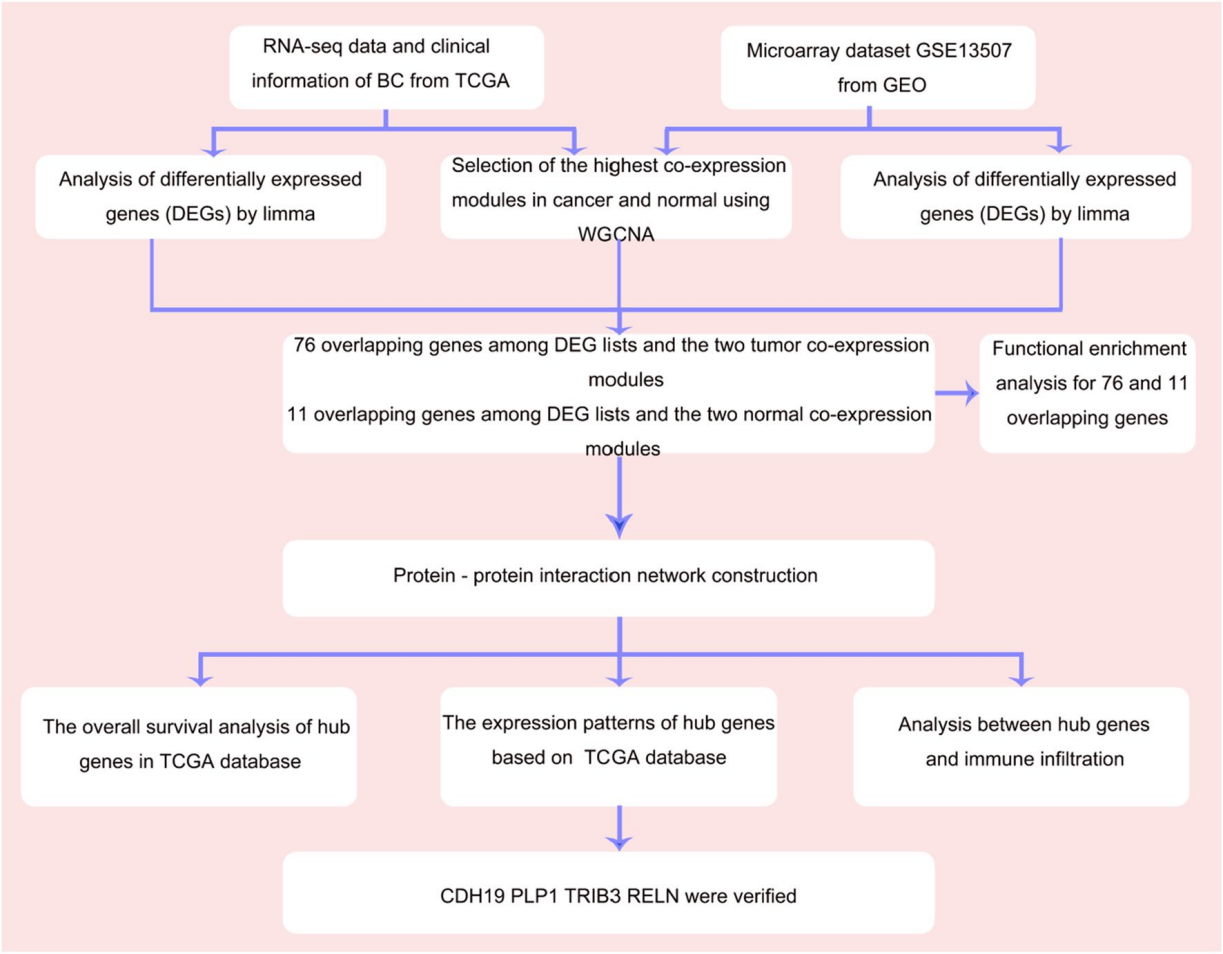
Workflow chart of bioinformatic analyses.

Hub gene extraction was performed strictly according to the flow chart shown in Fig. 1.

### Datasets from TCGA and GEO databases

RNA-sequencing profiles and clinical characteristics for patients with BC were obtained from TCGA (https://portal.gdc.cancer.gov/), employing the HTSeq-counts Workflow Type, and the GSE13507 dataset was downloaded from GEO (https://www.ncbi.nlm.nih.gov/gds). The TCGA (https://portal.gdc.cancer.gov/) dataset included 414 BC samples and 19 normal bladder tissues, and the RNAseq dataset included data for 19,601 genes. Genes with low read counts were excluded as per the instructions provided with the edgeR package^9^. Therefore, only genes with CPM (count per million) > 1 were included in subsequent analyses. A total of 14,830 genes with RPKM values were selected for further analysis using the RPKM function in the edgeR package.

Additionally, the GSE13507 dataset included data for 165 primary BC samples and 9 normal tissue samples obtained using the GPL6102 platform Illumina Human-6 v2.0 Expression Bead Chip. The dataset boasts a substantial sample size, coupled with comprehensive clinical data, thereby facilitating robust subsequent analyses. Probes were converted to gene symbols and duplicate probes for the same genes were removed via corresponding probes. Consequently, 24,324 genes were included in subsequent analyses.

### Differentially expressed genes identification

The R packages edgeR and limma were used for data collection and the identification of differentially expressed genes (DEGs) between tumour and normal tissues in the TCGA-BLCA and GSE13507 datasets. The DEGs were screened according to the following criteria: |logFC|> 1.0 and adjusted P < 0.05. The heatmap and ggplot2 packages were then used to visualize the DEGs and to generate a heatmap and volcano plot.

### Detection of key co-expression modules

Weighted co-expression networks were built with the WGCNA package in R using TCGA-BLCA and GSE13507 gene expression profiles. Notably, a signed network model was employed, encompassing both positive and negative correlations to capture nuanced gene co-expression patterns^10^. WGCNA identifies co-expression gene modules, relationships between gene networks and phenotypes, as well as core genes in networks. To establish a scale-free network and for soft thresholding power selection, pickSoft Threshold was used. Then, a_ij_ was calculated according to the following formula: a_ij =_ |S_ij_|^β^, where i and j represent genes, S_ij_ is the Pearson’s correlation coefficient, β is the soft thresholding power value, and a_ij_ indicates the adjacency coefficient. In this study, the optimal power value for the TCGA dataset is determined to be 3, while for the GEO dataset, the optimal power value is identified as 9. The strengths of the correlations for all intergenic expression levels were calculated, and an adjacency matrix was obtained. Furthermore, a topological overlap matrix (TOM) corresponding to the dissimilarity values (1-TOM) was generated. A hierarchical clustering tree was constructed using the correlation coefficients. Different branches in the clustering tree represent different gene modules (represented by different colours in the figures). Based on the weighted correlation coefficients, genes were classified according to expression patterns, and genes with similar patterns were classified into modules. Relationships between these modules and clinical parameters were evaluated to explore potential intergenic synergies related to tumorigenesis and development. The two modules with the highest positive correlation in the normal and tumour groups were selected for subsequent analyses using the TCGA-BLCA and GSE13507 datasets. The R package VennDiagram was used to evaluate overlapping DEGs between the two datasets and co-expressed genes in the two modules.

### Functional enrichment analysis of genes

The R packages cluster Profiler and enrich plot were used for Gene Ontology (GO) and Kyoto Encyclopaedia of Genes and Genomes (KEGG) pathway enrichment analyses of genes of interest^11^. GO uses ontologies to signify the biological relevance of interested genes by identifying overrepresented terms in three main categories: biological processes (BP), molecular functions (MF), and cellular components (CC)^12^. KEGG integrates genomic, chemical, and systemic functional information to connect genomes and biological processes^13^. Additionally, Perl calculated median gene expression for specified samples, generating GCT and CLS files for GSEA. GSEA 4.0.3 was used for analysis, followed by image integration in R.

### Construction of protein–protein interactions networks based on hub genes

To obtain a more elaborate and intuitive understanding of the protein interactions involving hub genes in the normal and tumour groups, the online tool, Search Tool for Retrieval of Interacting Genes (STRING, https://www.string-db.org/) was used to visualize hub genes and to construct a protein–protein interaction network, medium confidence is 0.4^14^.

### Validation of the prognostic value and expression of hub genes

To evaluate the clinical value of hub genes in the tumour and normal groups, TCGA and GEO data were used to detect the expression levels of these genes in BC and normal tissues. Similarly, the prognostic value of each hub gene was further evaluated using the survival package in R based on TCGA and GEO data. Only data with complete follow-up information were included in our study.

### Validation of hub gene protein expression

The expression levels of proteins encoded by hub genes were verified by immunohistochemical data in the Human Protein Atlas (HPA, https://www.proteinatlas.org/), which provides extensive transcriptome and proteome data and includes information on immunofluorescence localization^15^.

### Immune infiltration database

Tumour Immune Estimation Resource (TIMER), a database for evaluating the clinical effects of different immune cells in various tumours, was used to investigate the correlation between hub genes, copy number alterations, and immune infiltration levels. Additionally, the TISIDB (an integrated repository portal for tumor-immune system interactions) database was employed to investigate relationships between hub genes and immune infiltration therapy as well as subtypes (C5, immunologically quiet data were missing) in BC, and ENCORI (The Encyclopedia of RNA interactomes) was used for validation.

### Immunohistochemical for protein location and expression

The immunohistochemistry detection system kit (Rabbit) from Bioss antibodies, Cat. IHC001 was used to measure the expression of interested genes. Tissue chips of BC were obtained from Shanghai Outdo biotech company. The antibodies of hub genes were obtained from a Proteintech antibody supporter.

### Cell lines

Normal human bladder carcinoma epithelial cells SV-HUC-1, bladder carcinoma cell line 5637, and bladder transitional cell carcinoma T24, UM-UC-3, and SW780 were used in this study. All cells were obtained from Procell, Wuhan, China. SV-HUC-1, SW780, and UM-UC-3 cells were cultured using DMEM medium (Gibco, USA, Cat: 11965092) containing 10%FBS (Gibco, USA, Cat: 12483020) and 1% penicillin/streptomycin (Shanghai Beyotime Biotechnology, China), 5637 and T24 cells were cultured using FIM-1640 medium (Gibco, USA, Cat: 12633012) containing 10%FBS and 1% penicillin/streptomycin. All cells were cultured in a 37 °C aseptic incubator with 5% CO_2_.

### Protein collection and quantification

All cells were laid in 6-well plates with 5 * 10^6^ cells per well. After 24 h culture, the supernatant was discarded and cleaned twice with PBS. 200 μl RIPA cell lysate (Beijing Solebo, China, Cat: R0010) was added into each well, and the cell protein was fully recovered by gently blowing. The pyrolysis samples were centrifuged at 14000 rpm and 4 °C for 10 min to recover the supernatant. Then, the protein concentration was determined by a BCA protein concentration assay kit (Shanghai Yeasen Biotechnology Co., LTD., China, Cat: 20201ES76). 5% 2-mercaptoethanol in protein loading buffer was added in normalized protein for denaturation, then boiling at 100 °C for 5 min; proteins were stored at − 20 °C for further research.

### Western blot

30 µg of protein was separated on an 8–15% gel by SDS-PAGE and transferred to a PVDF membrane. After blocking in 5% bovine serum albumin in TBST for 1 h at room temperature, primary antibody CDH19 (China, Jiangsu, Affinity Biosciences, Cat.: DF3528), PLP1 (China, Jiangsu, Affinity Biosciences, Cat.: DF13282), TRIB3 (China, Jiangsu, Affinity Biosciences, Cat.: DF7844) was added and treated at 4℃ overnight. After washes with TBST, a goat anti-rabbit-HRP secondary antibody at a 1:20,000 dilution was incubated for 1 h at room temperature. The instruction GelView 6000 Pro from Photon Technology was used to reveal the blot, and the density of every blot was calculated by ImageJ.

### Quantitative PCR

The total RNA of five cells was isolated by cell RNA extraction kit (Shanghai Yeasen Biotechnology Co., China, Cat.: 19231ES50), then RNA was reverse transcribed using a reverse transcription kit (Shanghai Yeasen Biotechnology Co., China, Cat.: 11149ES60), qPCR was performed by QuantStudio 6Flex real-time fluorescent quantitative PCR (American, ThermoFisher) through the Hieff^®^ qPCR SYBR Green Master Mix (Shanghai Yeasen Biotechnology Co., China, Cat.: 11203ES50). The primer sequences of RELN were F: CAACCCCACCTACTACGTTCC, R: TCACCAGCAAGCCGTCAAAAA.

### Statistical analyses

Statistical analyses were performed using GraphPad software Prism 8.4.2. Data are expressed as mean with SEM. Protein quantification was all normalized to SV-HU-1. An unpaired t-test was used. *p < 0.05; **p < 0.01, ***p < 0.001; n.s. no significant difference.

### Equipment and settings

Immunohistochemical image were captured by confocal laser scanning electron microscopy (Zeiss, Germany). The images were exported by K-Viewer software.

As for western blot figures, the PVDF membrane containing interesting proteins were incubated in a chemiluminescence solution for 3 min, the blots were obtained by GelView6000 from Photo Technology. Due to the variability of the samples and antibodies used in each Western blot experiment, differences in both the exposure time and the results of the target strip were expected. The density of every blot was calculated by ImageJ software.

### Ethics statement

Ethical review and approval were not required for this study on human participants by the local legislation and institutional requirements, and neither the written informed consent for participation.

## Results

### Identification of significantly differentially expressed genes

**Figure 2:**
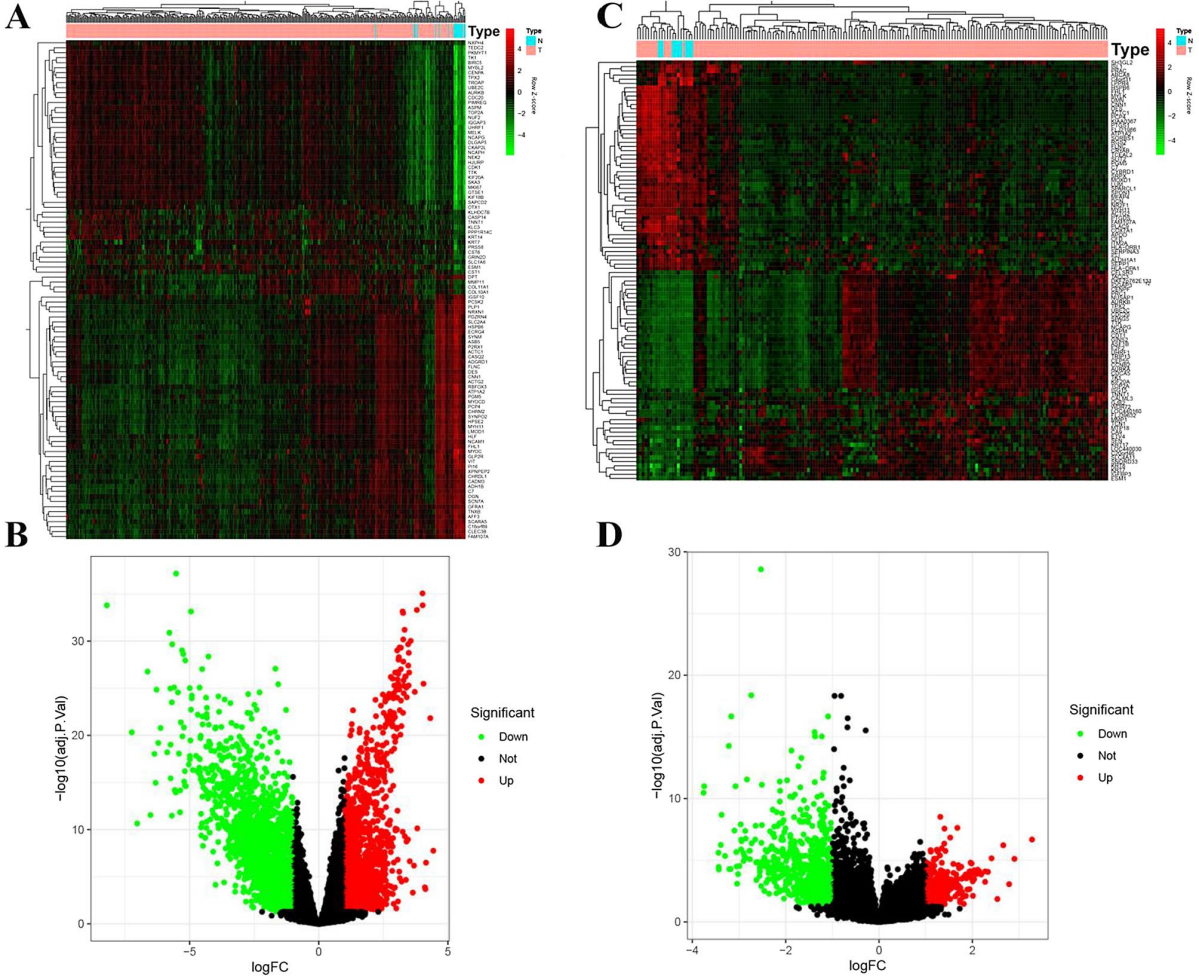
Identification of differentially expressed genes (DEGs) in the TCGA and GSE13507 bladder cancer datasets with the cut-off criteria: |logFC|> 1.0 and P < 0.05. (**A**) Heatmap of DEGs in the TCGA dataset. (**B**) Volcano plot of DEGs in the TCGA dataset. (**C**) Heatmap of DEGs in the GSE13507 dataset. (**D**) Volcano plot of DEGs in the GSE13507 dataset. T, tumor; N, normal samples.

Gene expression profiles for BC and para-cancerous tissues were obtained from the GEO and TCGA databases. After data filtering, normalisation, and ID transformation, two expression matrices were obtained with 414 BC samples and 19 adjacent bladder tissues in TCGA and 165 primary BC samples and 9 normal bladder tissues in GSE13507. The two datasets were used to screen for DEGs based on the established parameters. 3461 and 1069 DEGs met the screening criteria in TCGA-BLCA and GSE13507 datasets (Fig. 2).

### Weighted gene co-expression network construction

**Figure 3:**
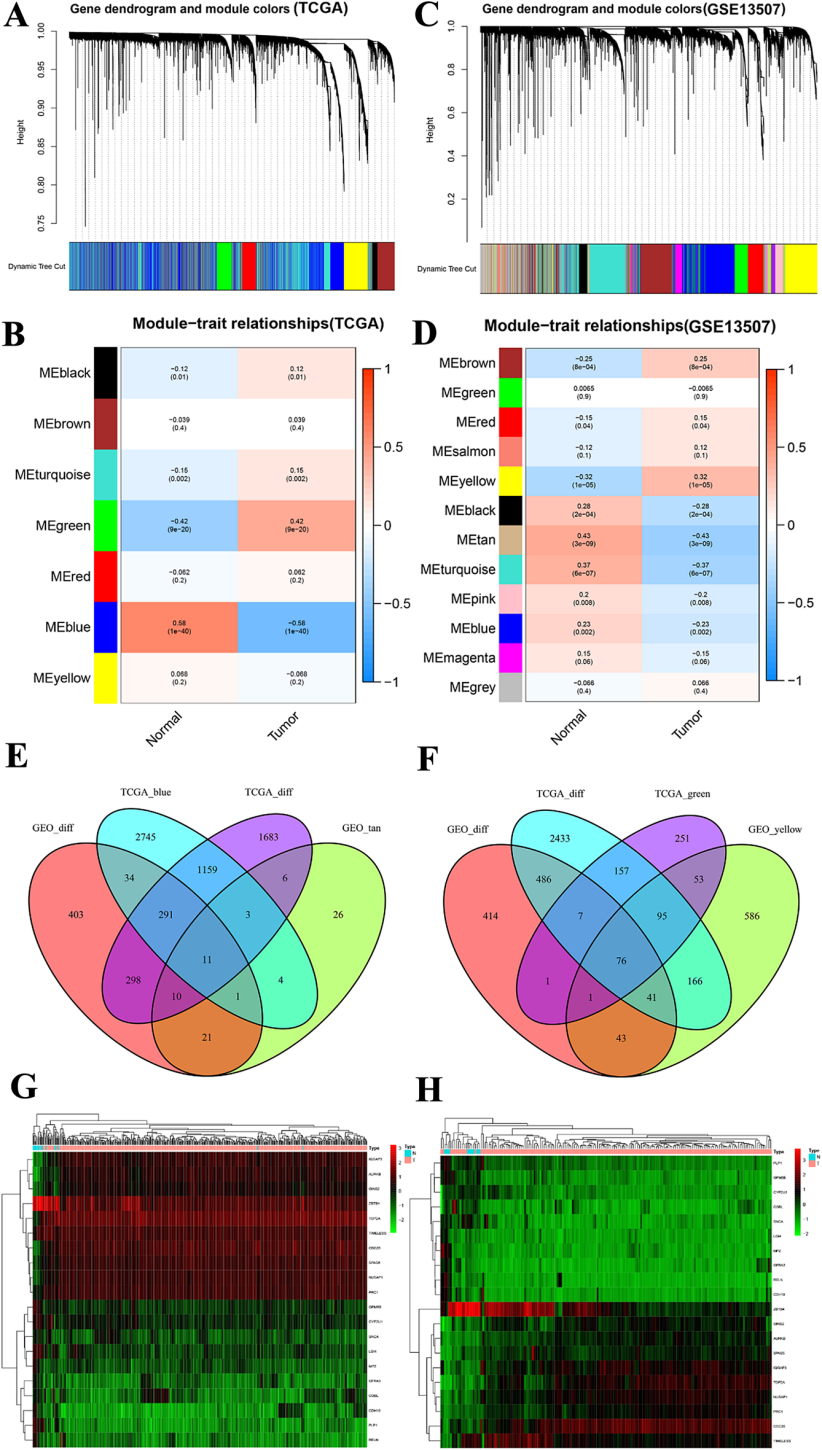
The relationship between modules and clinical features of bladder cancer in the TCGA-BC dataset (**A**,**B**) and GSE13507 dataset (**C**,**D**). (**A**) Gene clustering tree (dendrogram) based on hierarchical clustering of adjacency-based dissimilarity values. (**B**) Correlations between modules and clinical features (tumour and normal). (**C**) Gene clustering tree (dendrogram) based on hierarchical clustering of adjacency-based dissimilarity values. (**D**) Correlations between modules and clinical features (tumour and normal). Venn diagram of DEGs and genes in co-expression modules with differential expression in the normal group (**E**) and the tumour group (**F**). Expression heatmap of the top overlapping 20 genes in the TCGA dataset (**G**) and the GEO dataset (**H**).

To identify potential mechanisms underlying the development and progression of BC and to explore candidate biomarkers, the WGCNA package was used to construct a weighted gene co-expression network for both datasets (TCGA-BLCA and GSE13507). Seven gene modules were identified in TCGA-BLCA (Fig. 3A,B) and 12 gene modules were identified in the GSE13507 dataset (Fig. 3C,D, with different colours representing different gene modules). Subsequently, correlations between modules and clinical characters (tumour and normal) in TCGA-BLCA and GSE13507 were visualised by heatmaps (Fig. 3B,D). We identified modules that were highly associated with normal tissues (blue module in TCGA: *r* = 0.85, p < 1e−200; tan module in GSE13507: *r* = 0.64, p = 9.6e−11) and tumour tissues (green module in TCGA: *r* = 0.7, p = 1.7e−95; yellow module in GSE13507: *r* = 0.49, p = 3.6e−65). The overlap between genes in modules and DEGs in the two clinical groups (tumour and normal) was visualised by a Venn diagram. There were 11 overlapping genes in the normal group and 76 genes in the tumour group (Fig. 3E,F). These 87 overlapping genes were used for subsequent analyses. Expression heatmap of the top overlapping 20 genes in the TCGA dataset (Fig. 3G) and the GEO dataset (Fig. 3H).

### Functional annotation of overlapping DEGs

**Figure 4:**
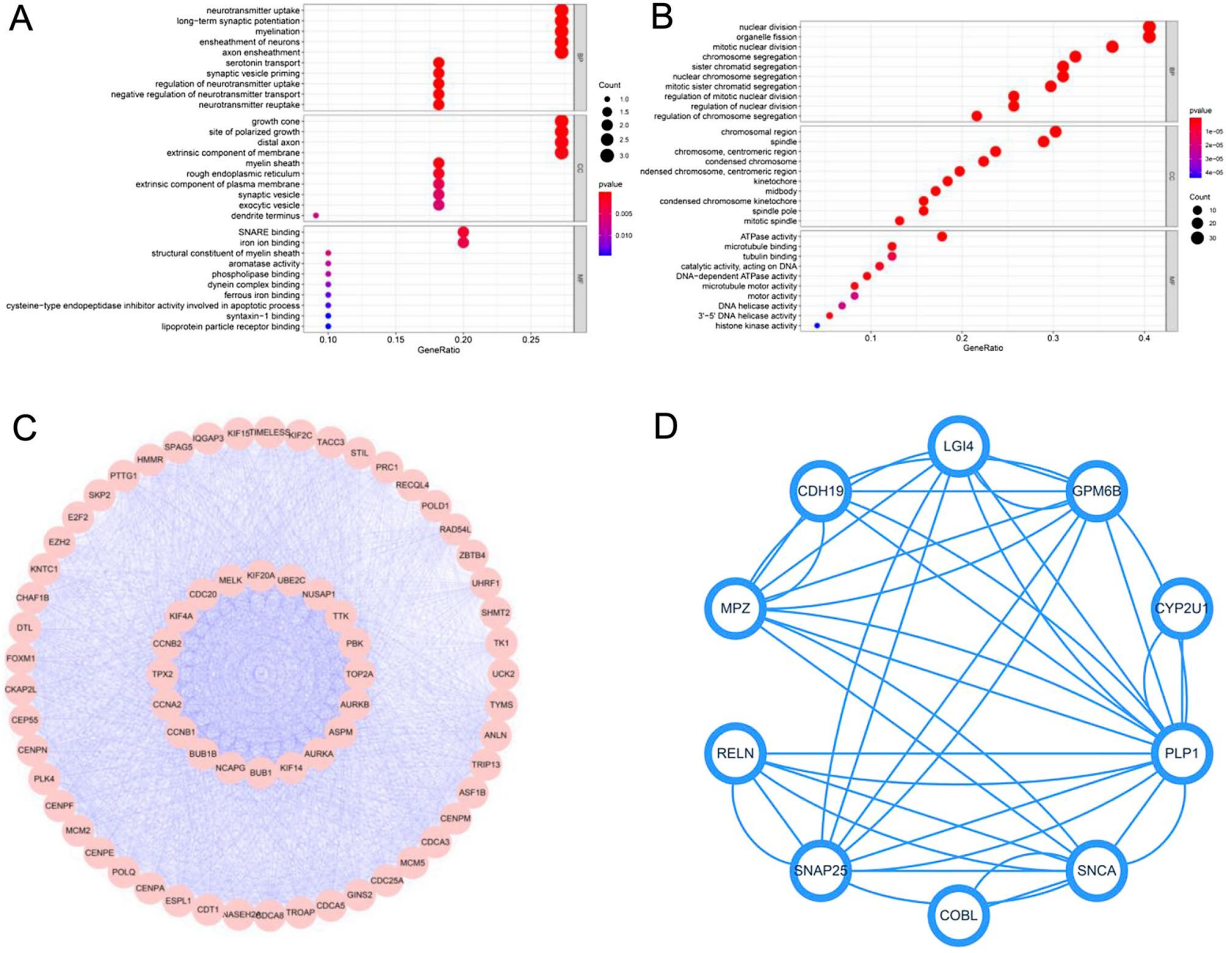
Significant modules based on a protein–protein interaction (PPI) network and Gene Ontology (GO) enrichment analysis. (**A**) GO enrichment analysis of hub genes in the normal group. (**B**) GO enrichment analysis of hub genes in the tumour group. (**C**) PPI network of hub genes in the tumour group. (**D**) PPI network of hub genes in the normal group.

The potential functions of 11 overlapping genes in the normal group and 76 genes in the tumour group were explored by GO and KEGG pathway enrichment analyses. As shown in Fig. 4A,B, in the BP category, 11 overlapping genes in the normal group were mainly enriched in neurotransmitter uptake, long-term synaptic potentiation, and serotonin transport (Fig. 4A), and 76 genes in the tumour group were mainly related to mitotic nuclear division, nuclear division, sister chromatid segregation, and regulation of chromosome segregation (Fig. 4B), which are important processes in the development of tumours. In the CC category, overlapping genes in the normal group were related to growth cone and the site of polarised growth, and genes in the tumour group were related to chromosomal regions, condensed chromosomes, centromeric regions, and condensed chromosome kinetochores. In the MF category, genes in the normal groups were mainly involved in soluble N-ethylmaleimide-sensitive factor attachment protein receptor (SNARE) binding and iron ion binding, and overlapping genes in the tumour group were mainly enriched in ATPase activity and 3′ to 5′ DNA helicase activity. A KEGG pathway enrichment analysis showed that intersecting genes in the normal group were linked to arachidonic acid metabolism and synaptic vesicle cycle and those in the tumour group were related to mitotic nuclear division, regulation of mitotic nuclear division, and metaphase/anaphase transition of the fiumitotic cell cycle (Fig. S1).

### PPI network construction and hub gene identification

PPI networks for the normal and tumour groups were constructed using the STRING database (Fig. 4C,D), and the CytoHubba plugin was then used to calculate scores based on the MCC algorithm to select hub genes. As shown in Fig. 4C, the top 20 genes with the highest scores in the tumour group, including *KIF2C, NUSAP1, MELK, PBK, KIF20A, AURKA, NCAPG, TPX2, KIF4A, ASPM, AURKB, CDC20, CCNB1, BUB1B, CCNB2, CCNA2, BUB1, TOP2A, UBE2C,* and *TTK,* and the top 11 genes with the highest scores in the normal group, including GPM6B, *CYP2U1, SNCA, PLP1, LGI4, RELN, MPZ, CDH19, GFRA3, COBL*, and *SNAP25,* were identified as hub genes.

### Screening and validation of prognostic markers involves assessing the expression of proteins encoded by hub genes

**Figure 5:**
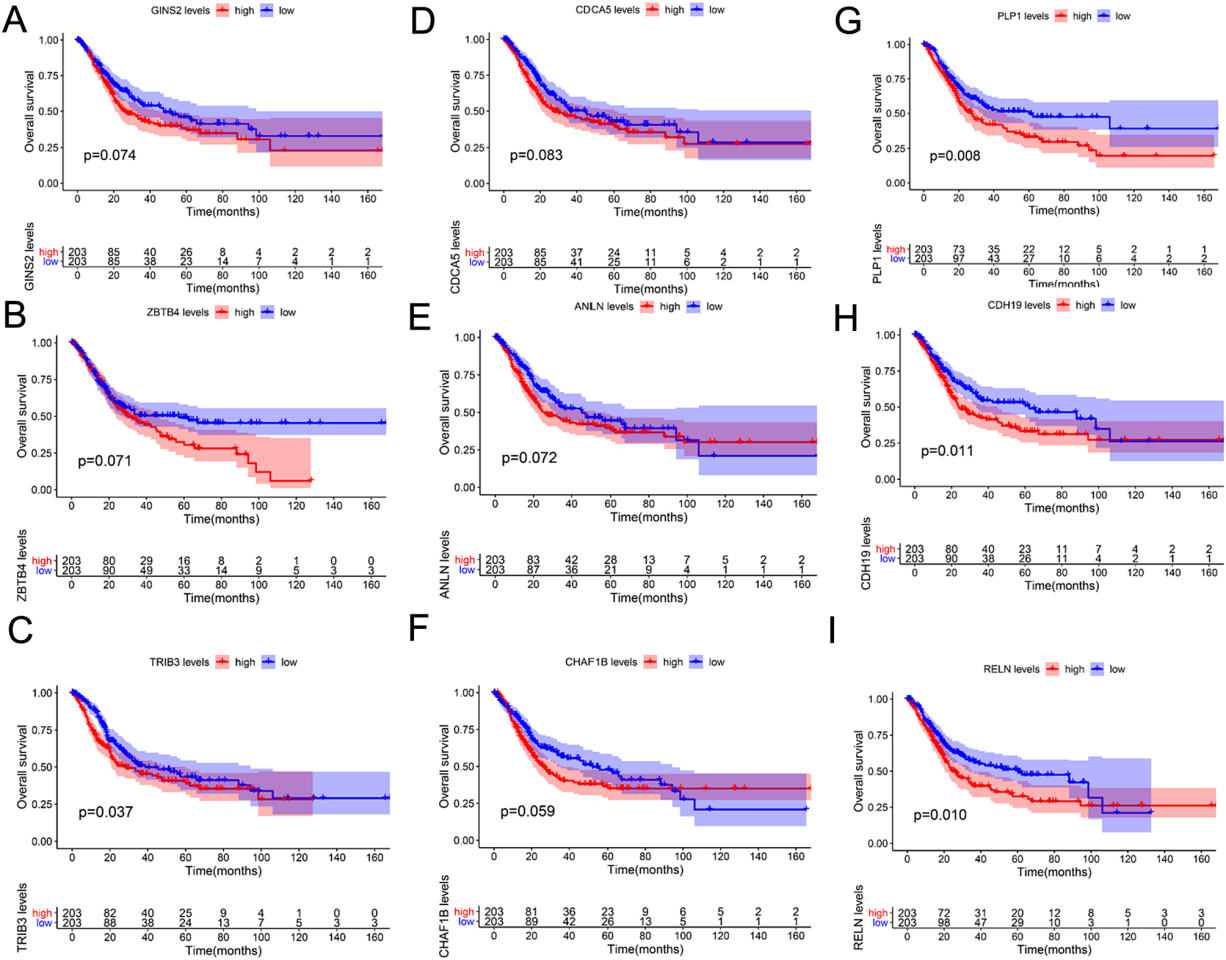
Validation of the prognostic value of hub genes (**A**) GINS2. (**B**) ZBTB4. (**C**) TRIB3. (**D**) CDCA5. (**E**) ANLN. (**F**) CHAF1B. (**G**) PLP1. (**H**) CDH19. (**I**) RELN.

To gain further insight into the prognostic value of hub genes, the survival package in R was used to analyse 31 hub genes in both normal and tumour groups. Nine genes were associated with the prognosis of BC (Fig. 5, Fig. S3E–H), with four being significantly associated with prognosis, i.e., *CDH19, RELN, PLP1,* and *TRIB3″* (P < 0.05). Moreover, these four hub genes were closely related to the clinical stage of BC (Fig. S2). The expression levels of the four hub genes were verified in the TCGA-BLCA dataset and the GEO dataset (Figs. S3A,D, S4), *CDH19, RELN*, and *PLP1* levels in normal bladder tissues were significantly higher than those in BC tissues, while the expression of *TRIB3* was higher in BC tissues than in normal bladder tissues. Furthermore, the protein expression level of TRIB3 was explored using the HPA online database (http://www.proteinatlas.org/). As shown in Fig. S5, the expression of TRIB3 in normal bladder tissues was higher than that in tumour tissues, contrary to the results for RNA expression.

### Identification of hub gene-related pathways by a gene set enrichment analysis

**Figure 6:**
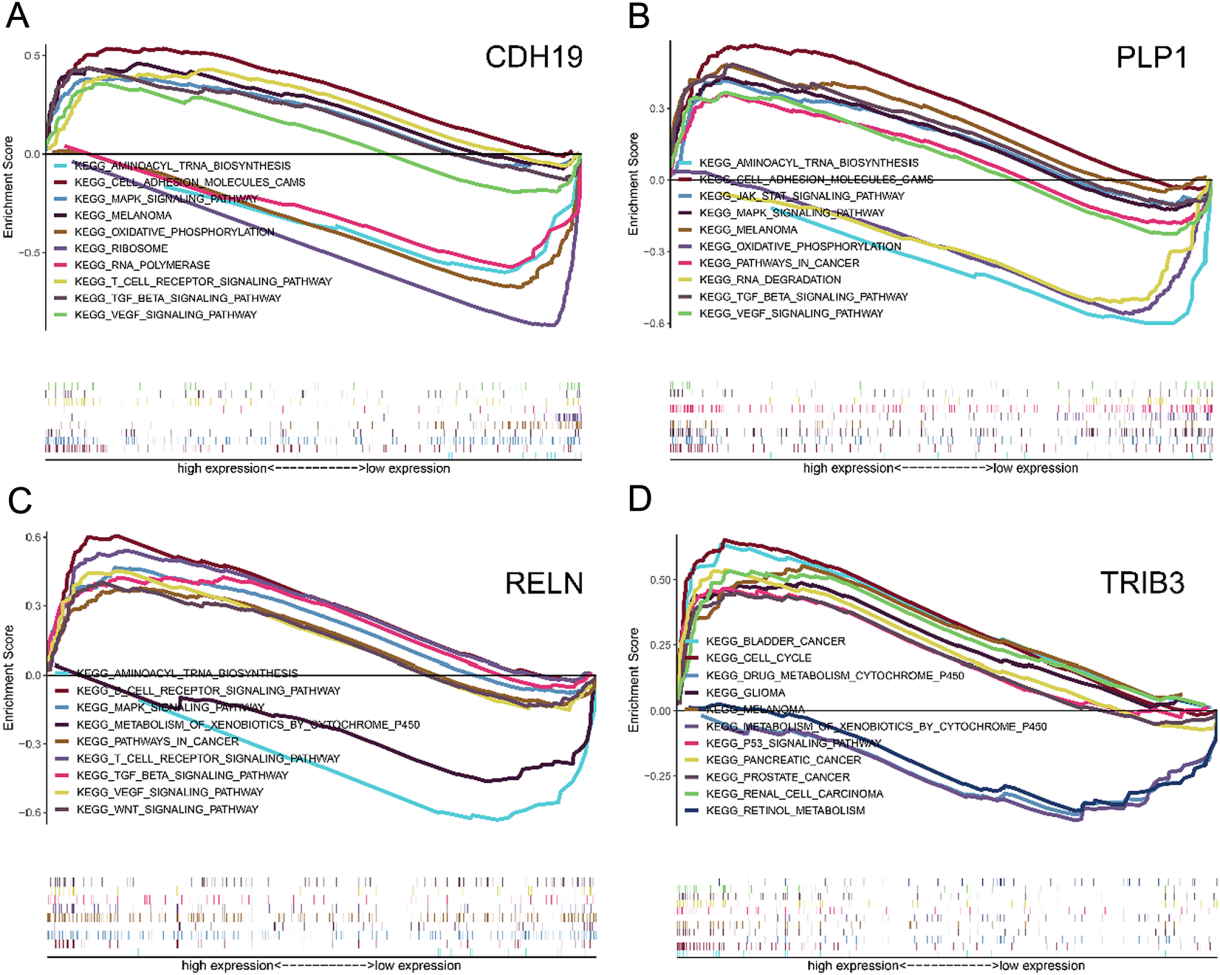
Identification of hub genes-related pathways associated with the prognosis of bladder cancer determined by a multiple gene set enrichment analysis (MGSEA), functional enrichment analyses of different expression levels. (**A**) Pathways enriched analysis at high and low CDH19 expression group; (**B**) Pathways enriched analysis at high and low PLP1 expression group; (**C**) Pathways enriched analysis at high and low RELN expression group; (**D**) Pathways enriched analysis at high and low TRIB3 expression group.

Pathways involving four hub genes in BC were evaluated by a GSEA. The expression levels of hub genes were categorised as high and low following the cutoff value for hub genes. Then, GSEA was performed using two datasets for four hub genes based on the normalised enrichment score (NES), nominal P-value (NOM P-value), and false discovery rate (FDR). The enrichment results for the MSigDB gene sets were significantly different according to the GSEA results (NOM P-val < 0.05, FDR < 0.05). As shown in Fig. 6A, the MAPK, VEGF, and cell adhesion molecule pathways were significantly enriched in the CDH19-related phenotype. The cell adhesion molecules, MAPK signalling pathway, JAK-STAT signalling pathway, and cancer-related pathways were significantly enriched in the PLP1-related phenotype (Fig. 6B). The WNT signalling pathway, cancer-related pathways, VEGF signalling pathway, and MAPK signalling pathway were significantly enriched in the RELN-related phenotype (Fig. 6C). Moreover, TRIB3 was significantly related to BC, cell cycle, and P53 signalling pathways (Fig. 6D).

### Associations between hub genes and immune infiltration or immune therapy

The relationships between hub genes and immune cell infiltration were explored using the TIMER algorithm (http://cistrome.dfci.harvard.edu/TIMER/)^16^. As shown in Fig. S6 and supplemental online Table 1, multiple types of copy number alterations in the four hub genes, especially deletions, were significantly related to immune cells in BC, and the deletion of hub genes can reduce the level of immune infiltration in multiple immune cells. Negative correlations were detected, as shown in Fig. S7, between hub genes and tumour purity (*CDH19*, *r* = − 0.26, p = 4.29e−07; *PLP1*, *r* = − 0.311, p = 9.63E−10; *RELN*, *r* = − 0.382, p = 3.06e−14; *TRIB3*, *r* = − 0.138, p = 8.14e−03). The statistical significance and Spearman’s correlation coefficients for the relationships between *RENL, PLP1, TRIBE, CDH19*, and immune cell infiltration signatures are shown in supplemental online Tables 1 and 2. The immune cell signatures included CD8+ T cells, T cells (general), B cells, monocytes, tumour-associated macrophages (TAMs), M1 macrophages, M2 macrophages, macrophages, neutrophils, natural killer cells, dendritic cells, Th1, Th2, Tfh, Th17, Treg, and T cell exhaustion (*p < 0.05; **p < 0.01; ***p < 0.001; ****p < 0.0001). Furthermore, the TISIDB database^17^ was used to explore the value of hub genes in BC immunotherapy. We divided BC into six groups (wound healing, IFN-gamma-dominant, inflammatory, lymphocyte-depleted, immunologically quiet, and TGF-β-dominant) according to the classification described by Thorsson et al.^18^. We found that TRIB3 expression was the highest in the IFN-gamma-dominant subtype, CDH19 expression was highest in wound healing, and both PLP1 and RELN levels were high in the inflammatory type (Fig. S8). Additionally, using thresholds of |r|> 0.5 and p < 0.05, we found that both PLP1 and RELN were significantly associated with CXCL12 (Fig. S9A, PLP1, *r* = 0.542, p < 2.2e−16; RELN, *r* = 0.513, p < 2.2e−16), an immunoinhibitory factor. In addition, RELN was also strongly related to ENTPD1 (Fig. S9B, *r* = 0.497, p < 2.2e−16). The relationships between *PLP1, RELN, ENTPD1,* and *CXCL12* were verified using the ENCORI database (Fig.S9D–F), which yielded consistent results.

### *CDH19* might be associated with BC formation

**Figure 7:**
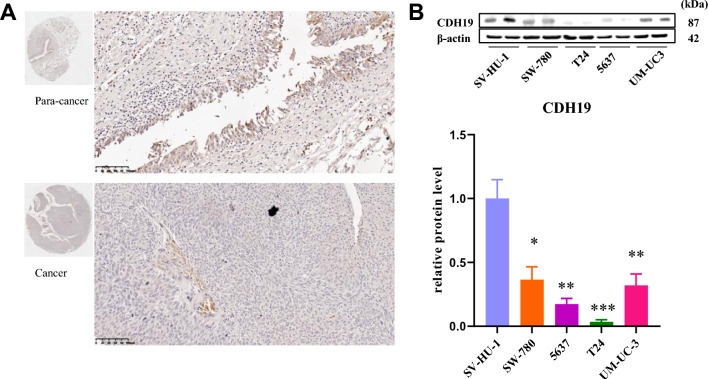
(**A**) CDH19 showed higher protein expression in bladder paracancer tissues compared with cancer tissues. (**B**) Protein expression (up) and quantification (down) of CDH19 in different bladder cancer cell lines. An unpaired t-test was used. *p < 0.05; **p < 0.01; ***p < 0.001; n.s. no significant difference, more than three times experiment have been launched (n > 3). Original gels are presented in Supplementary Figure expanded view 1.

According to the data analyses online, we launched immunohistochemistry for CDH19 utilizing a tissue chip containing 25 paired cancer and para-cancer tissues, we found out only a few amounts of CDH19 expression in BC tissues contrast with the corresponding para-cancer tissues (Fig. 7A). We measured the translation level of CDH19 by western blot in the BC cell lines mentioned above, it turns out the protein level of CDH19 decreased according to the increase of BC grade (Fig. 7B). Above all, we give our hypothesis that CDH19 might involve in BC progression and be a candidate target for treatment in the future.

### *PLP1* might involve in cytokines metabolism in BC tissues

PLP1 protein expression is increased only in cell line 5637 which constitutively produces and secretes several functionally active cytokines. This cell is a valuable, reliable, and inexpensive source for the culture of growth factor-responsive^19^. On the contrary, TRIB3 protein was inhibited in this cell line (Fig. S10A,B).

## Discussion

BC is a malignant tumour with high morbidity and mortality; the overall prognosis of BC has been poor. Compared with MIBC, the prognosis of NMIBC is much better; however, approximately two-thirds of cases show superficial BC relapse, despite significant progress in diagnosis and treatment. Approximately 30% of recurrent cases in bladder cancer are expected to progress to muscle-invasive bladder cancer (MIBC), with a subset of patients demonstrating distant metastases, contributing to a suboptimal 5-year survival rate^20^. Therefore, early diagnosis and treatment of BC are necessary.

In this study, hub genes associated with the development of BC were identified and characterised (i.e., *KIF2C, NUSAP1, MELK, PBK, KIF20A, AURKA, NCAPG, TPX2, KIF4A, ASPM, AURKB, CDC20, CCNB1, BUB1B, CCNB2, CCNA2, BUB1, TOP2A, UBE2C, TTK, TRIB3, GPM6B, CYP2U1, SNCA, PLP1, LGI4, RELN, MPZ, CDH19, GFRA3, COBL*, and *SNAP25*). GO and KEGG pathway enrichment analyses indicated that hub genes in the normal group were mainly enriched in neurotransmitter uptake, long-term synaptic potentiation, and serotonin transport, and those biology processes in the tumour group were associated with cell proliferation. Among these genes, four genes related to prognosis, *CDH19, RELN, PLP1*, and *TRIB3*, were further identified. These four hub genes were significantly enriched in the MAPK signalling pathway, VEGF signalling pathway, WNT signalling pathway, cell cycle, and P53 signalling pathway based on a gene set enrichment analysis (Fig. 6). In addition, these genes were also associated with immune infiltration levels in BC and showed different expression characteristics in BC cell lines (Fig. S6).

*CDH19*, *RELN*, and *PLP1* expression levels in normal bladder tissues were significantly higher than those in BC tissues, while the expression of *TRIB3* was higher in BC tissues than in normal bladder tissues. *TRIB3* inhibits Akt/PKB activation by insulin in liver^21^. Several studies reveal that TRIB3 promoting the progression of lung cancer, clear cell renal cell carcinoma, colorectal cancer and acute myeloid leukaemia^22–26^, however, few is known about the regulation of *TRIB3* in BC. Nevertheless, the pattern of TRIB3 protein expression based on data in the HPA online database was contrary to the mRNA expression pattern, so further studies are needed to evaluate this interesting phenomenon.

Subsequently, a GSEA was performed to identify enriched pathways. As shown in Fig. 6, *CDH19*, *PLP1*, and *RELN* were both involved in the MAPK signalling pathway. MAPK, including two major isoforms, p38α (MAPK14) and p38β, is associated with the local inflammatory cascade in peripheral tissues^27^. *PLP1* is often related to nucleotide substitutions and copy number variation, leading to Pelizaeus-Merzbacher disease^28^. *PLP1* is also involved in cancer-related pathways and the JAK-STAT signalling pathway, and participant in tumorigenesis, maintenance, and metastasis in breast cancer^29^. The role of PLP1 in BC has not been determined yet and requires further study. Furthermore, as determined using the TIMER algorithm, various mutation types in the four hub genes, especially deletions, were significantly associated with immune cells in BC, and the deletion of hub genes can reduce the level of immune infiltration in multiple immune cell types (supplemental online Tables 1 and 2). Additionally, we found that levels of both *PLP1* and *RELN* were significantly associated with levels of *CXCL12*, which plays an important role in numerous physiological and pathological processes, including tumour metastasis^30^. *RELN* is associated with the WNT signalling pathway (Fig. 6), which plays a significant role in initiation, progression, and metastasis in various types of cancers^31^. As for *RELN*, we only performed a transcription test for which the results showed that RELN also decreased along with the increased grade of BC. *RELN* also shows high expression in the SW780 cell line (Fig. S11). After multiple repeated experiments, RELN detection by western blotting was hindered, possibly due to its excessive molecular weight. SW780 is used to create a cell line-derived xenograft mouse model, and enables the study of BC treatment. SW780 is a grade I carcinoma cell line, which means that *RELN* could become a pre-diagnosis for bladder carcinomas. As shown in the results, *PLP1* and *TRIB3* were characterized by an inverse expression profile in 5637 cell line. Are *PLP1* and *TRIB3* playing two panels in a balance in cytokines mechanism in BC? It will be worth to investigate the function of those two genes in our perspective work.

*CDH19* is a cadherin type II gene situated on chromosome 18^32^. Previous research has revealed that loss of cadherins may be connected with tumour formation. Cadherins are considered prime candidates for tumour suppressor genes. The down-regulation of *CDH19* might support putative involvement in head and neck cancer progression^33^. SV-HUC-1 represents an immortalized healthy human ureteral epithelial cell line. T24 cells originated from a high-grade urinary bladder cancer patient in 1973, 5637 cells were derived from a grade II bladder carcinoma, and SW780 cells were established in 1974 from a grade I transitional cell carcinoma. The UM-UC-3 cell line's grade remains unknown. In our investigation, a progressive diminution in CDH19 protein expression was observed concomitant with the advancing stages of BC. This trend implies a potential association between reduced CDH19 levels and the pathogenesis of BC, underscoring CDH19 as a promising candidate for prognostic assessments in the context of bladder cancer. A meticulous exploration of the implicated biological processes and molecular mechanisms governing cancer development is imperative for comprehensive understanding and warrants further investigation.To conclude, combining our comprehensive bioinformatics analysis with scientific research experiments, our study enabled the identification of four candidates for potential prognostic value of BC and found out the tip of the iceberg function of *CDH19, PLP1, TRIB3*, and *RELN*. Our finding pointed to a few research orientations for those proteins in BC. The molecular mechanisms and effectiveness of these genes in BC are worth to be evaluated further.

### Supplementary Information

Supplementary Information.

Supplementary Figures.

Supplementary Table 1.

Supplementary Table 2.

## Data Availability

RNA-sequencing profiles and clinical characteristics for patients with BC were obtained from TCGA (https://portal.gdc.cancer.gov/), and the GSE13507 dataset was downloaded from GEO (https://www.ncbi.nlm.nih.gov/gds).

## References

[1] Siegel RL, Miller KD, Jemal A (2020). Cancer statistics, 2020. CA Cancer J. Clin..

[2] Ghervan L, Zaharie A, Ene B (2017). Small-cell carcinoma of the urinary bladder: Where do we stand?. Clujul Med..

[3] Karakiewicz PI, Benayoun S, Zippe C (2006). Institutional variability in the accuracy of urinary cytology for predicting recurrence of transitional cell carcinoma of the bladder. BJU Int..

[4] Jacobs BL, Lee CT, Montie JE (2010). Bladder cancer in 2010: How far have we come?. CA Cancer J. Clin..

[5] Ploeg M, Aben KK, Kiemeney LA (2009). The present and future burden of urinary bladder cancer in the world. World J. Urol..

[6] Zhang B, Horvath S (2005). A general framework for weighted gene co-expression network analysis. Stat. Appl. Genet. Mol. Biol..

[7] Yip AM, Horvath S (2007). Gene network interconnectedness and the generalized topological overlap measure. BMC Bioinform..

[8] Segundo-Val IS, Sanz-Lozano CS (2016). Introduction to the gene expression analysis. Methods Mol. Biol..

[9] Robinson MD, Mccarthy DJ, Smyth GK (2010). edgeR: A Bioconductor package for differential expression analysis of digital gene expression data. Bioinformatics.

[10] Langfelder P, Horvath S (2008). WGCNA: An R package for weighted correlation network analysis. BMC Bioinform..

[11] Yu G, Wang LG, Han Y (2012). clusterProfiler: An R package for comparing biological themes among gene clusters. OMICS.

[12] The Gene Ontology C (2017). Expansion of the Gene Ontology knowledgebase and resources. Nucleic Acids Res..

[13] Kanehisa M, Sato Y, Furumichi M (2019). New approach for understanding genome variations in KEGG. Nucleic Acids Res..

[14] Szklarczyk D, Gable AL, Lyon D (2019). STRING v11: Protein-protein association networks with increased coverage, supporting functional discovery in genome-wide experimental datasets. Nucleic Acids Res..

[15] Maity B, Sheff D, Fisher RA (2013). Immunostaining: Detection of signaling protein location in tissues, cells and subcellular compartments. Methods Cell Biol..

[16] Li T, Fan J, Wang B (2017). TIMER: A web server for comprehensive analysis of tumor-infiltrating immune cells. Cancer Res.

[17] Ru B, Wong CN, Tong Y (2019). TISIDB: An integrated repository portal for tumor-immune system interactions. Bioinformatics.

[18] Thorsson V, Gibbs DL, Brown SD (2019). The immune landscape of cancer. Immunity.

[19] Quentmeier H, Zaborski M, Drexler HG (1997). The human bladder carcinoma cell line 5637 constitutively secretes functional cytokines. Leukemia Res..

[20] Kamat AM, Hahn NM, Efstathiou JA (2016). Bladder cancer. Lancet.

[21] Du K, Herzig S, Kulkarni RN (2003). TRB3: A tribbles homolog that inhibits Akt/PKB activation by insulin in liver. Science.

[22] Wu XQ, Tian X, Xu FJ (2022). Increased expression of tribbles homolog 3 predicts poor prognosis and correlates with tumor immunity in clear cell renal cell carcinoma: A bioinformatics study. Bioengineered.

[23] Hua F, Mu R, Liu J (2011). TRB3 interacts with SMAD3 promoting tumor cell migration and invasion. J. Cell Sci..

[24] Liu C, Zhang W, Wang J (2021). Tumor-associated macrophage-derived transforming growth factor-β promotes colorectal cancer progression through HIF1-TRIB3 signaling. Cancer Sci..

[25] Luo X, Zhong L, Yu L (2020). TRIB3 destabilizes tumor suppressor PPARα expression through ubiquitin-mediated proteasome degradation in acute myeloid leukemia. Life Sci..

[26] Zhou W, Ma J, Meng L (2022). Deletion of TRIB3 disrupts the tumor progression induced by integrin αvβ3 in lung cancer. BMC Cancer.

[27] Hale KK, Trollinger D, Rihanek M (1999). Differential expression and activation of p38 mitogen-activated protein kinase alpha, beta, gamma, and delta in inflammatory cell lineages. J. Immunol..

[28] Bahrambeigi V, Song X, Sperle K (2019). Distinct patterns of complex rearrangements and a mutational signature of microhomeology are frequently observed in PLP1 copy number gain structural variants. Genome Med..

[29] Shao F, Pang X, Baeg GH (2020). Targeting the JAK/STAT signaling pathway for breast cancer. Curr. Med. Chem..

[30] Li J, Chen H, Zhang D (2021). The role of stromal cell-derived factor 1 on cartilage development and disease. Osteoarthr. Cartil..

[31] Imani A, Maleki N, Bohlouli S (2020). Molecular mechanisms of anticancer effect of rutin. Phytother. Res..

[32] Shao F, Pang X, Baeg GH (2021). Targeting the JAK/STAT signaling pathway for breast cancer. Curr. Med. Chem..

[33] Blons H, Laccourreye O, Houllier AM (2002). Delineation and candidate gene mutation screening of the 18q22 minimal region of deletion in head and neck squamous cell carcinoma. Oncogene.

