# Supplementary material for: Identification and preliminary analysis of hub genes associated with bladder cancer progression by comprehensive bioinformatics analysis
Source: Sci Rep. 2024 Feb 2;14:2782. doi: 10.1038/s41598-024-53265-z (PMC10837156; doi:10.1038/s41598-024-53265-z)
Supplement: Supplementary file 1 — Supplementary Information. [file 41598_2024_53265_MOESM1_ESM.pdf]

Figure Expanded View 1. The order of loaded samples was the same as in the upper figure 6B.

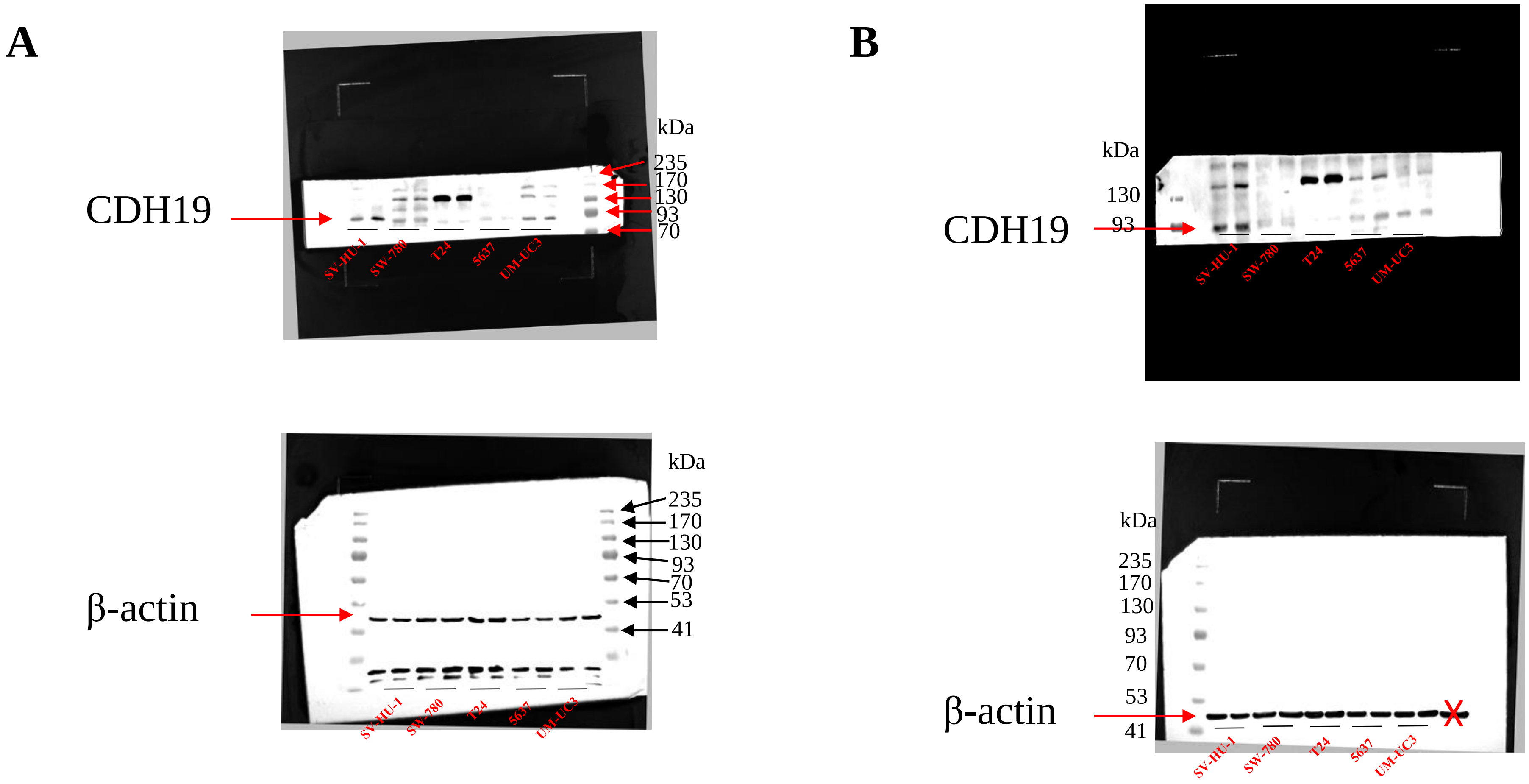

Figure EV1: (A, B) The detection of CDH19 and β-actin was the result of exposure of the same cell samples on different membranes. The proteins of bladder cancer cells SV-HU-1, SW-780, T24, 5637, and UM-UC3 were used for WB detection in the same amounts.

Figure Expanded View 2. The order of loaded samples was the same as in upper Figure S10A and Figure S10B.

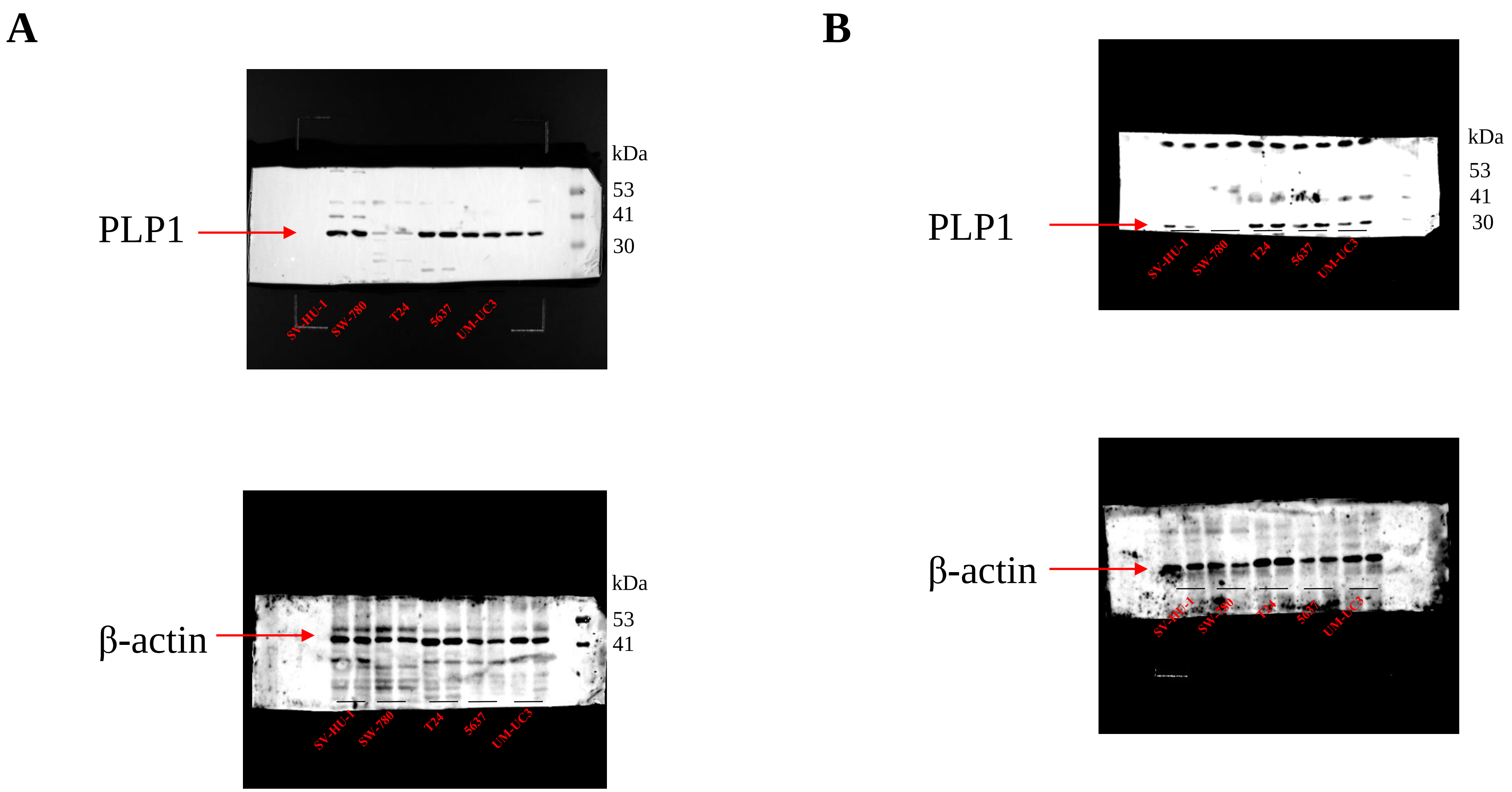

Figure EV2: (A, B) The detection of PLP1and β-actin was the result of exposure with the same samples on the same membrane. The proteins of bladder cancer cells SV-HU-1, SW-780, T24, 5637, and UM-UC3 were used for WB detection in the same amounts.

Figure Expanded View 3. The order of loaded samples was the same as in upper Figure S10A or Figure S10B.

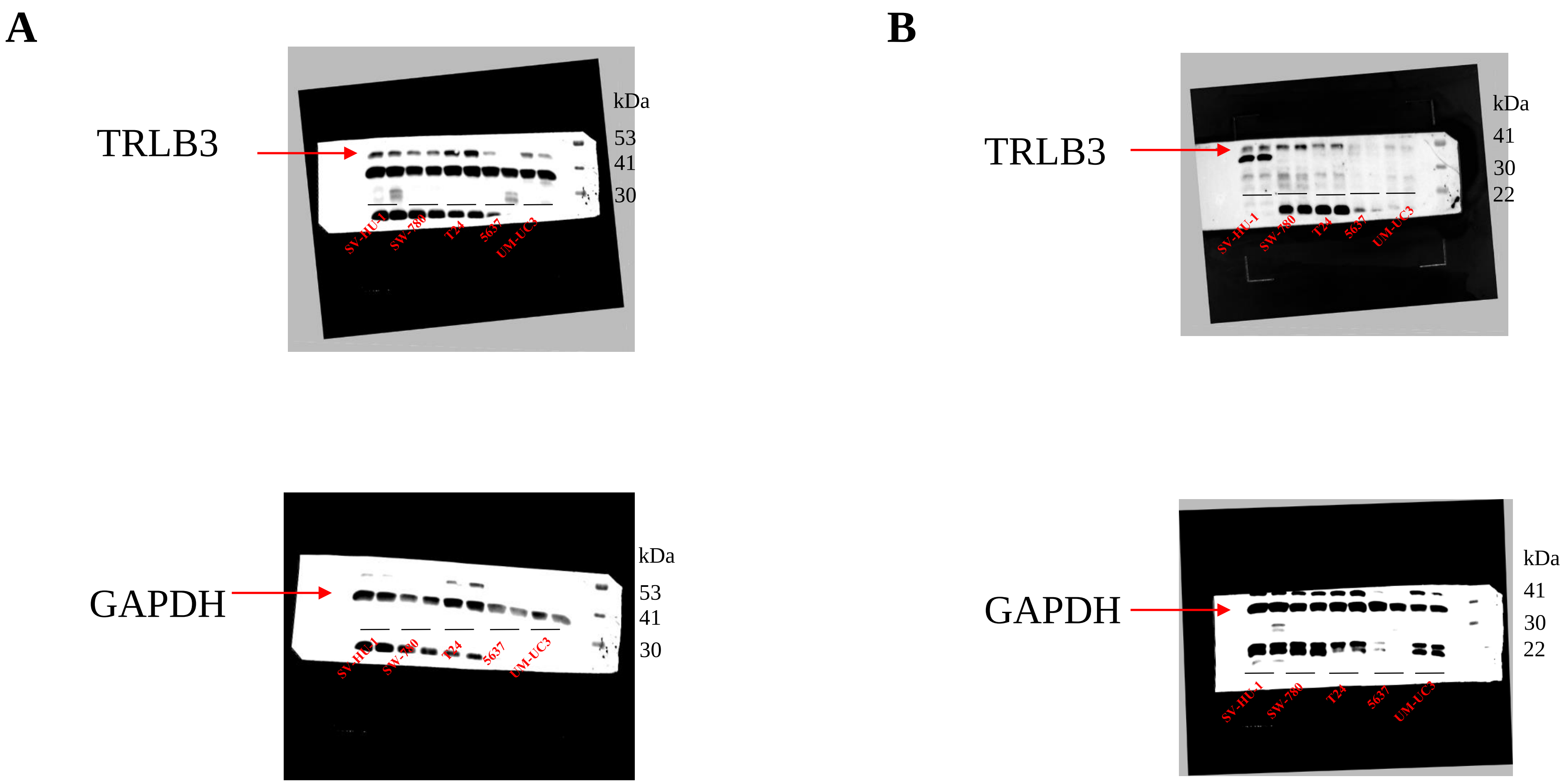

Figure EV3: (A) The detection of TRLB3 and GAPDH were launched using the same sample on the same membrane; (B) The detection of TRLB3 and GAPDH was performed in different membranes individually. The proteins of bladder cancer cells SV-HU-1, SW-780, T24, 5637, and UM-UC3 were used for WB detection in the same amounts.
