# Supplementary material for: Identification and preliminary analysis of hub genes associated with bladder cancer progression by comprehensive bioinformatics analysis
Source: Sci Rep. 2024 Feb 2;14:2782. doi: 10.1038/s41598-024-53265-z (PMC10837156; doi:10.1038/s41598-024-53265-z)
Supplement: Supplementary file 2 — Supplementary Figures. [file 41598_2024_53265_MOESM2_ESM.pdf]

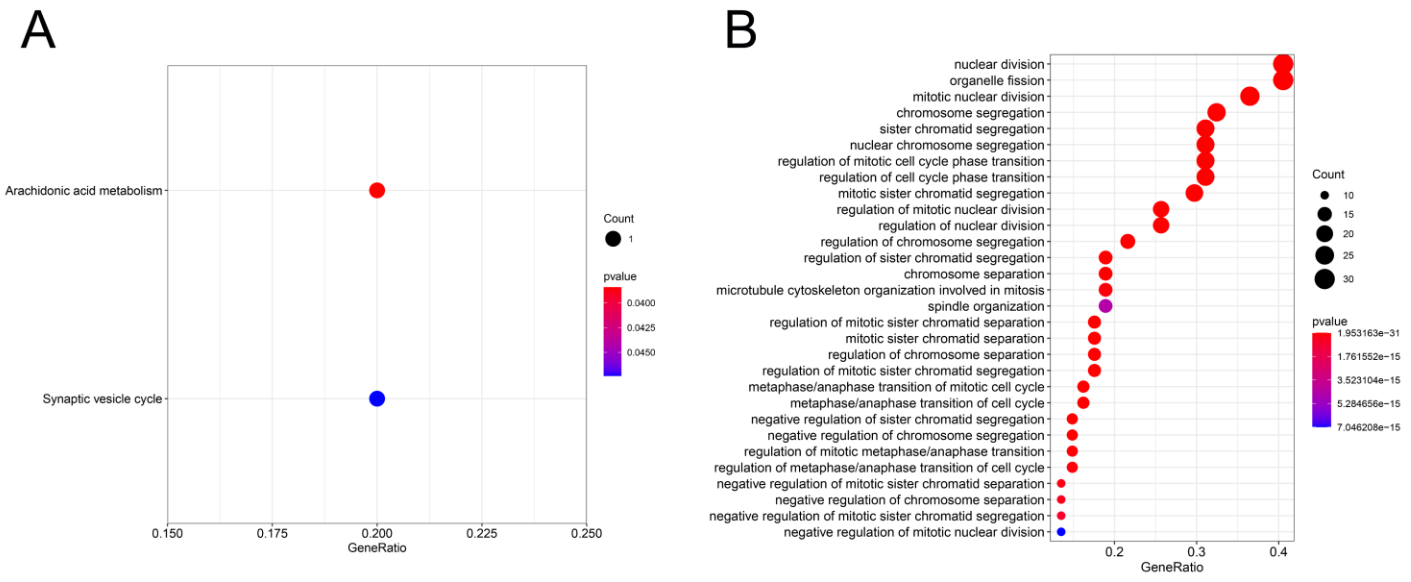

Figure S1: (A) Kyoto Encyclopedia of Genes and Genomes (KEGG) enrichment analysis of hub genes in the normal group. (B) KEGG enrichment analysis of hub genes in the cancer group.

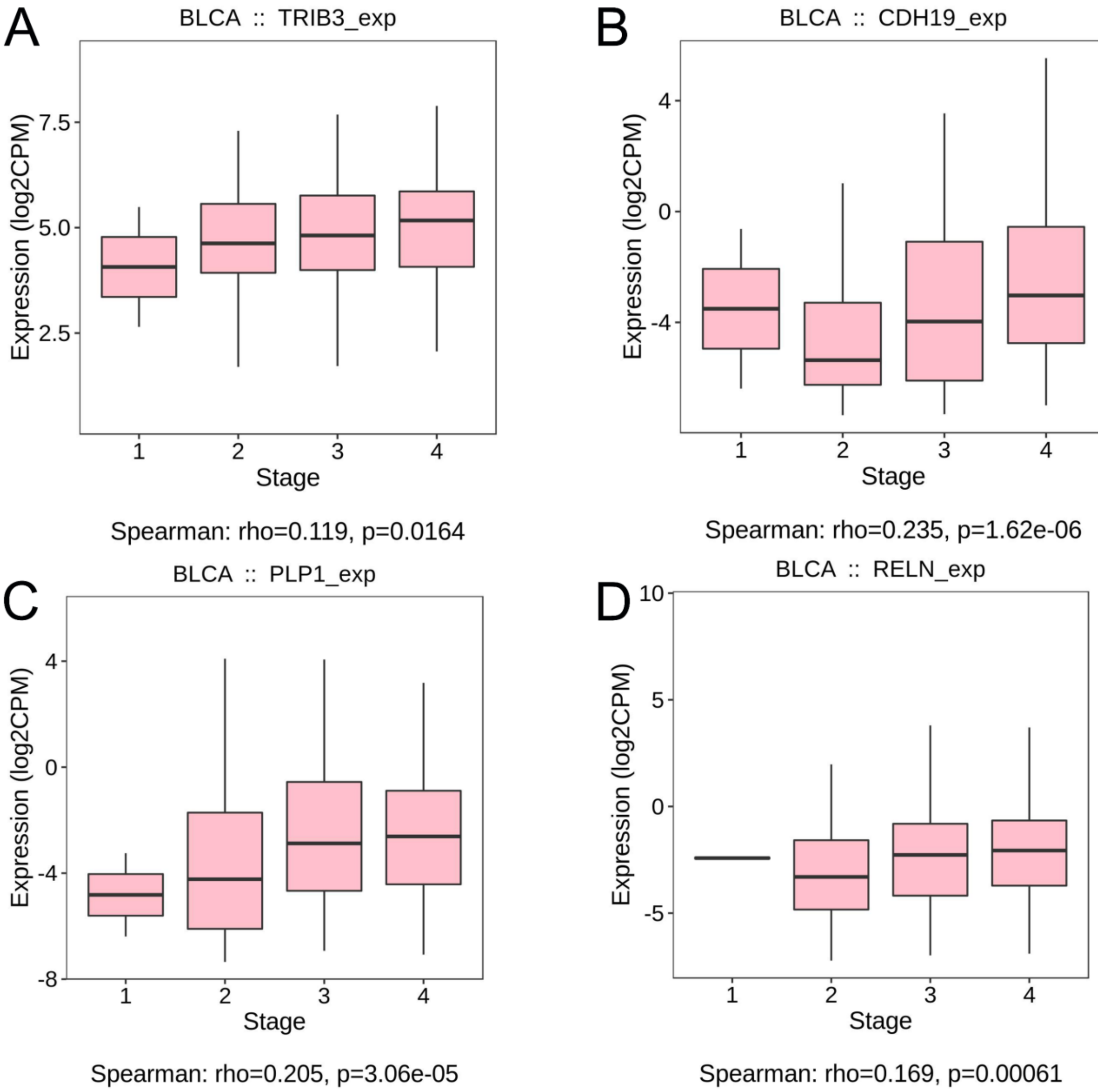

Figure S2: Correlations between expression levels of four hub genes and clinical stage. (A) *TRIB3*; (B) *CDH19*; (C) *PLP1*; (D) *RELN*.

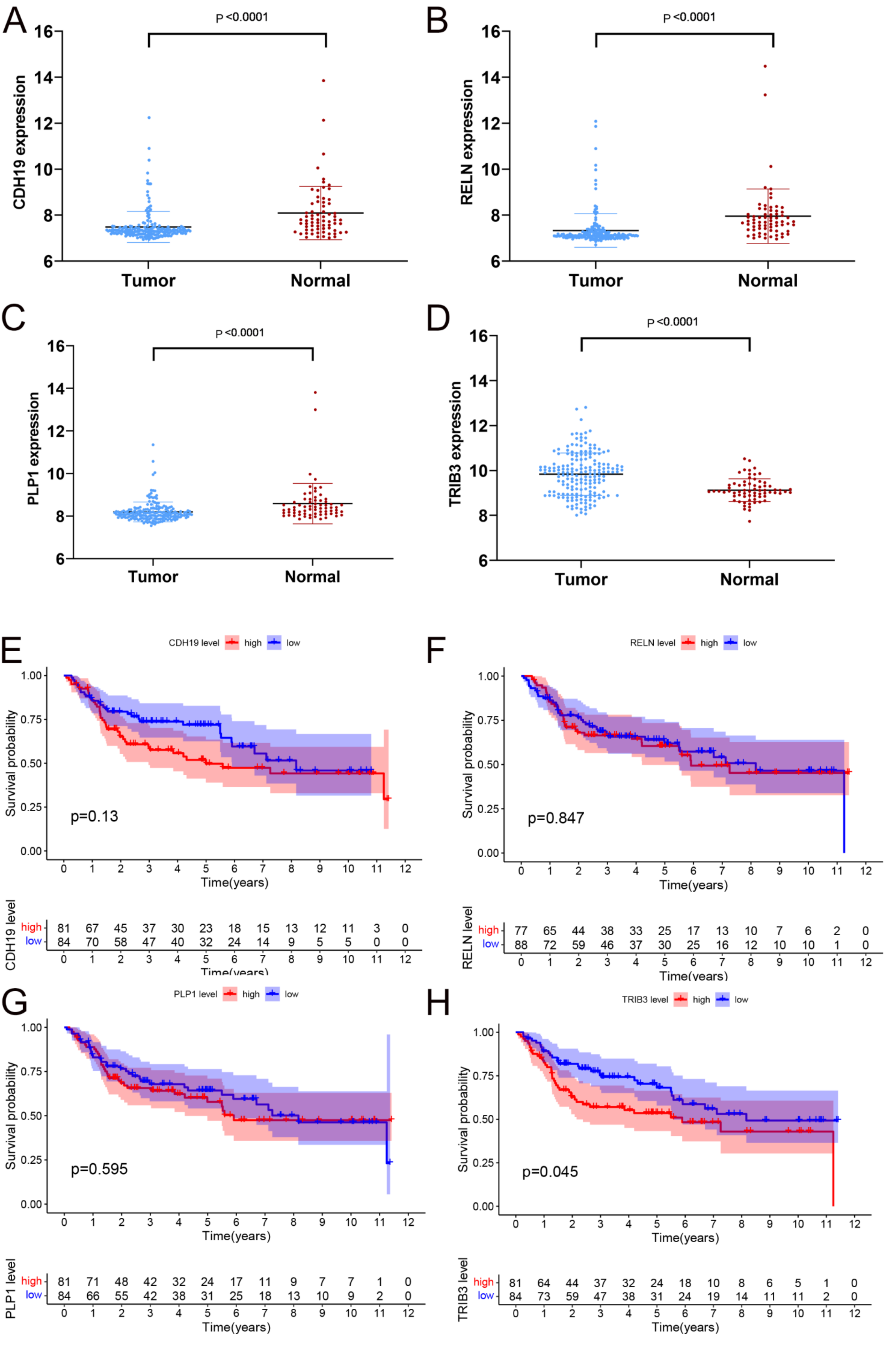

Figure S3: Expression validation and prognostic value analysis in bladder cancer of four hub genes in the GEO dataset. (A, E) *CDH19*; (B, F) *RELN*; (C, G) *PLP1*; (D, H) *TRIB3*.

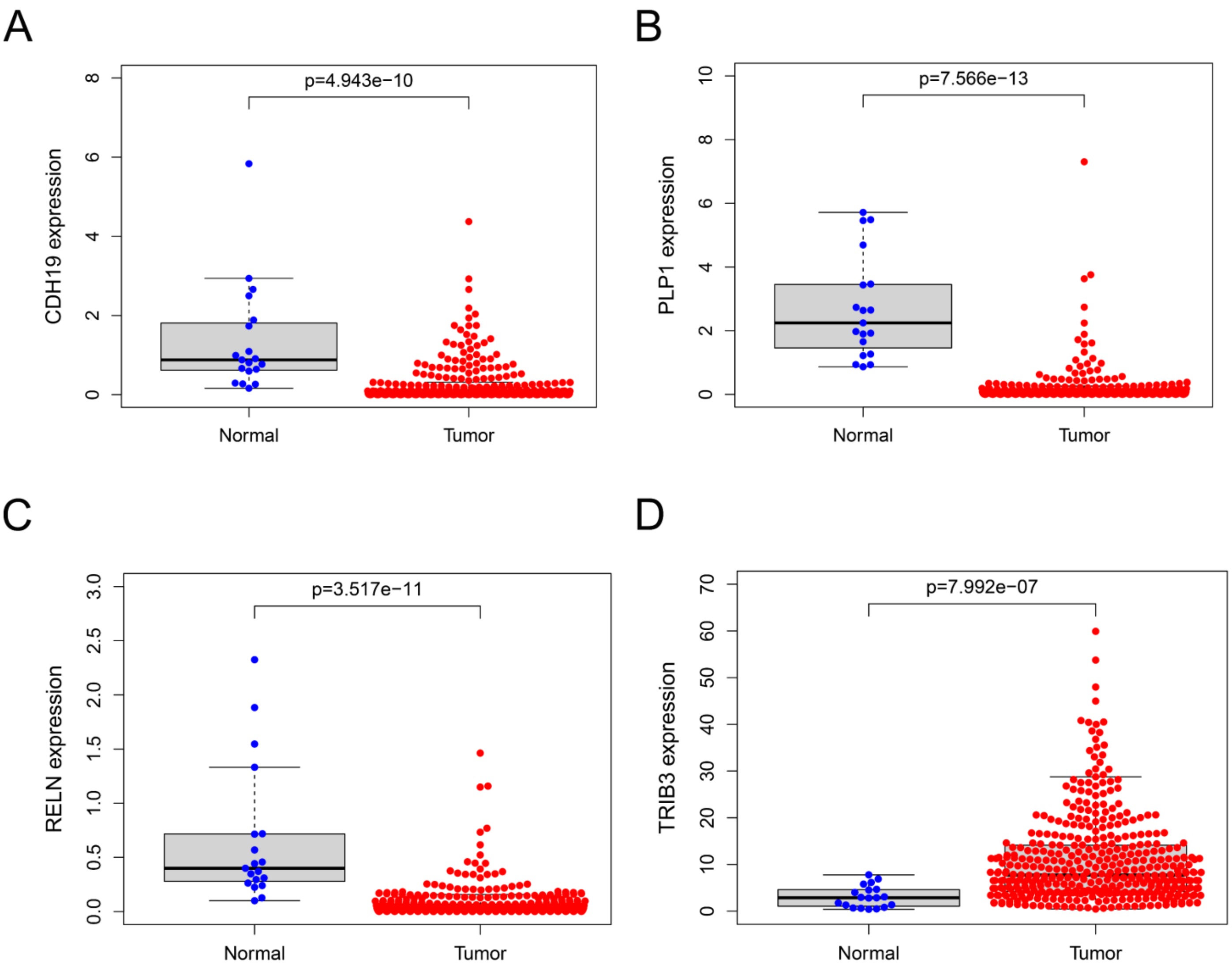

Figure S4: The expression of hub genes associated with the prognosis of bladder cancer. (A) *CDH19*; (B) *PLP1*; (C) *RELN*; (D) *TRIB3*.

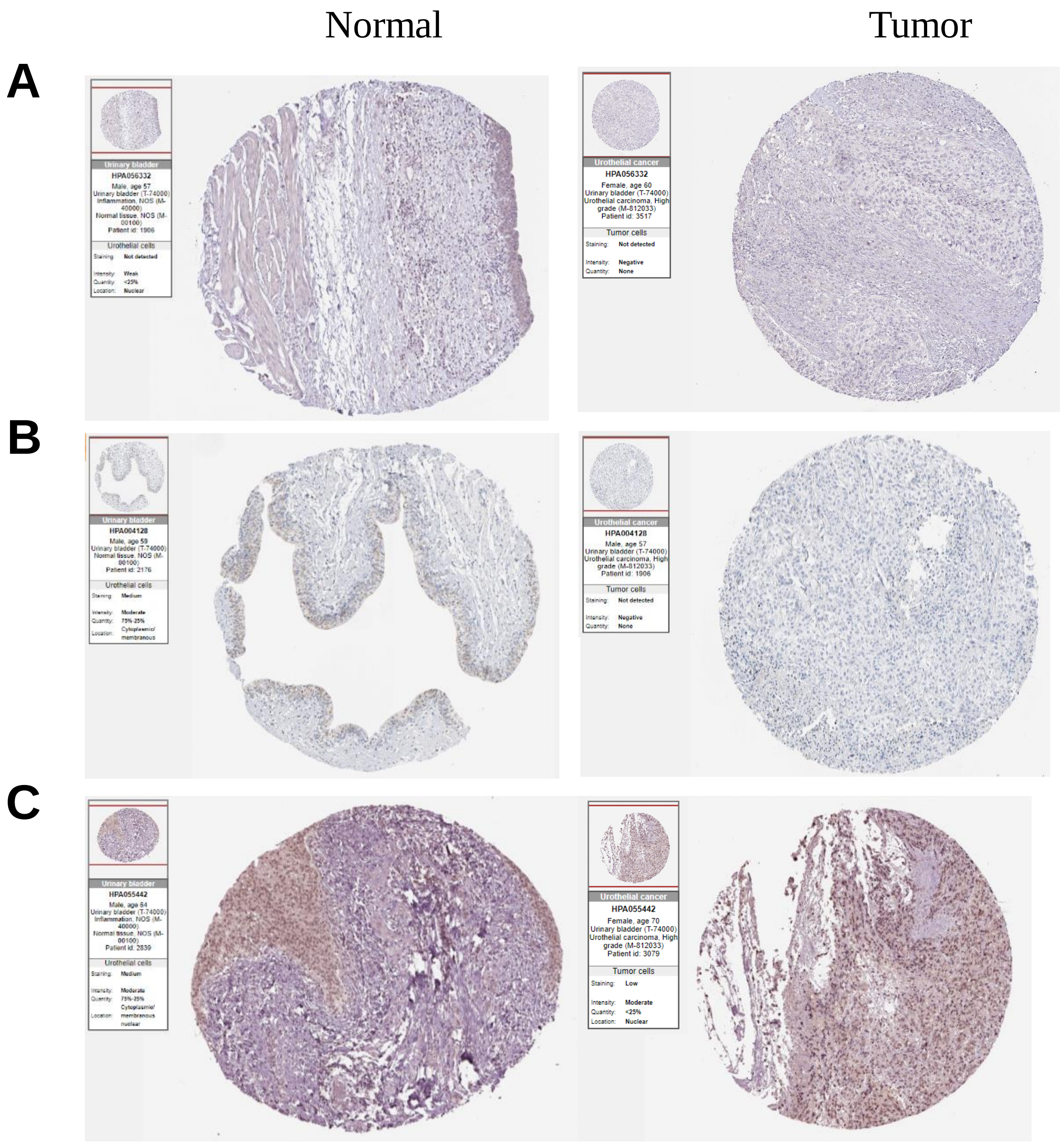

Figure S5: Immunohistochemistry of TRIB3 in normal tissues and bladder cancer from the Human Protein Atlas (HPA). (A) Protein levels of CDH19 in normal tissues (left) and cancer tissues(right). (B) Protein levels of PLP1 in normal tissues (left) and cancer tissues(right). (C) Protein levels of TRIB3 in normal tissues (left) and cancer tissues(right). There is no IHC data for RELN in HPA database.

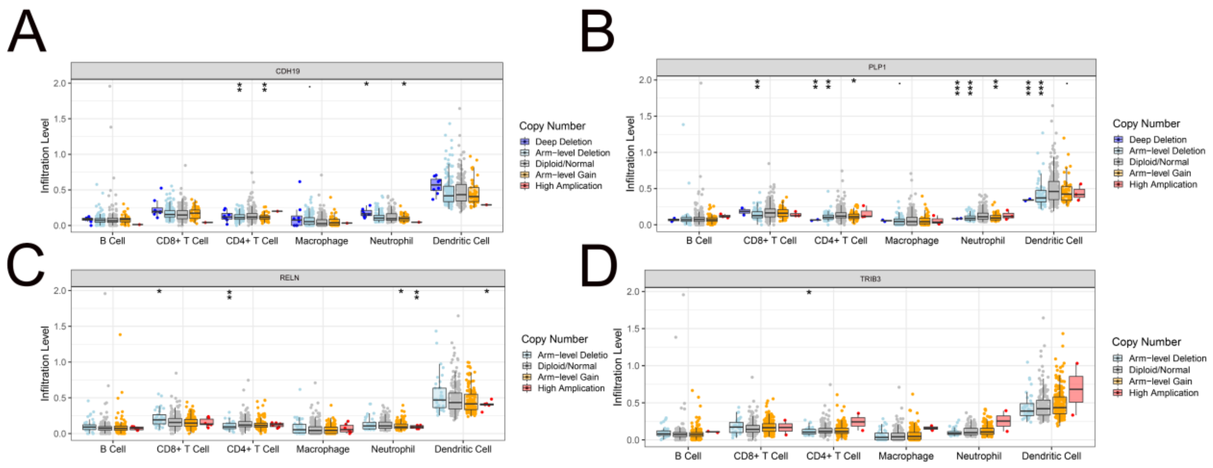

Figure S6: Effect of genetic alterations in hub genes on immune cell infiltration. (A) *CDH19*; (B) *PLP1*; (C) *RELN*; (D) *TRIB3*.

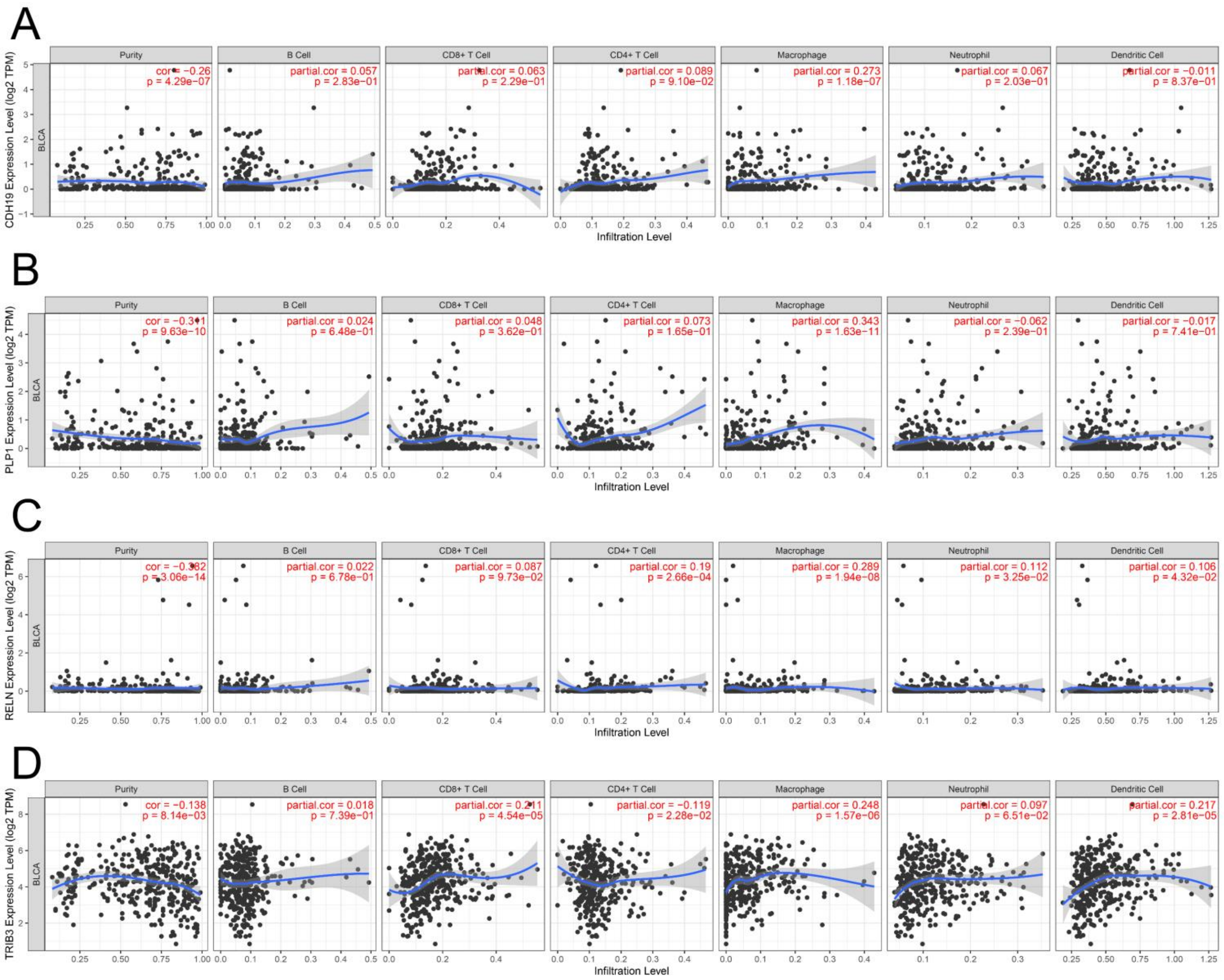

Figure S7: Correlations between expression levels of four hub genes and six types of immune cells, as well as the tumor purity in the TIMER database. (A) *CDH19*; (B) *PLP1*; (C) *RELN*; (D) *TRIB3*

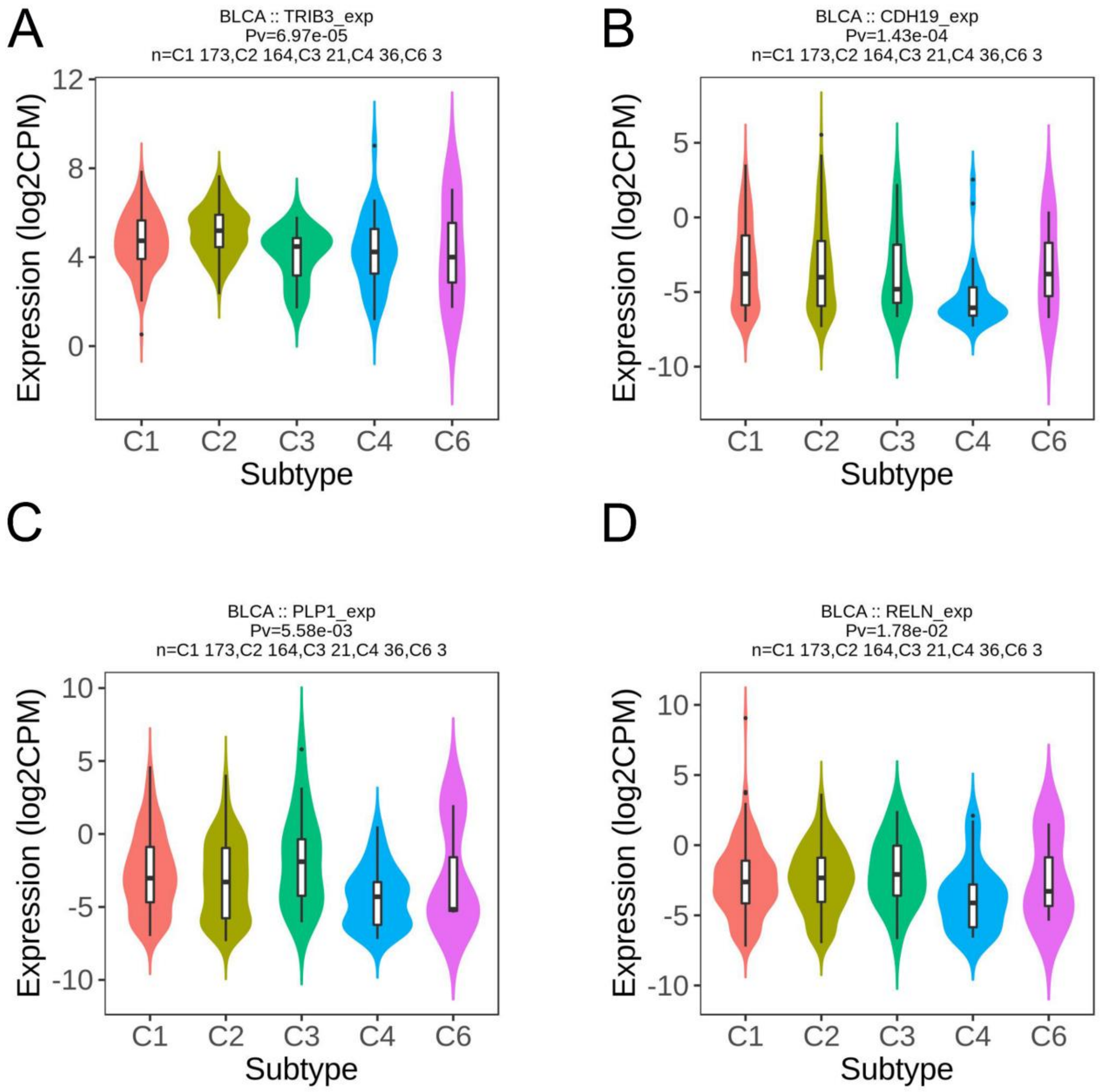

Figure S8: Expression of hub genes in different immune subtypes of bladder cancer (A) *TRIB3*; (B) *CDH19*; (C) *PLP1*; (D) *RELN*. (C1, wound healing ; C2, IFN-gamma-dominant ;C3, inflammatory; C4, lymphocyte-depleted ; C6, TGF-β-dominant)

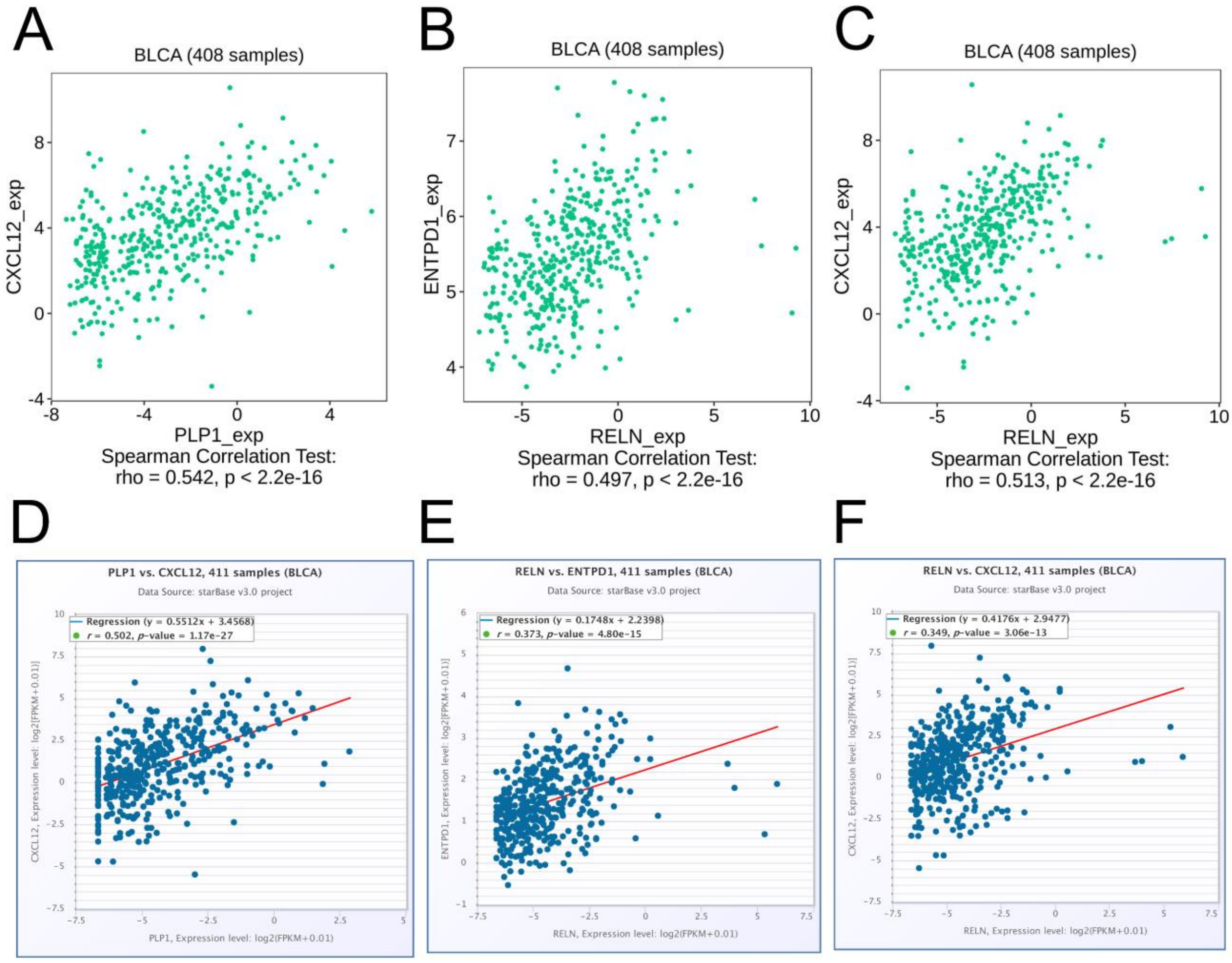

Figure S9: Scatterplots of correlations between RELN, PLP1 levels and CXCL12, ENTPD1 levels based on filter standards (|r| > 0.5 and $p < 0.05$) (A–C); Validation of correlations between RELN, PLP1 expression and CXCL12, ENTPD1 using starBase (D–F)

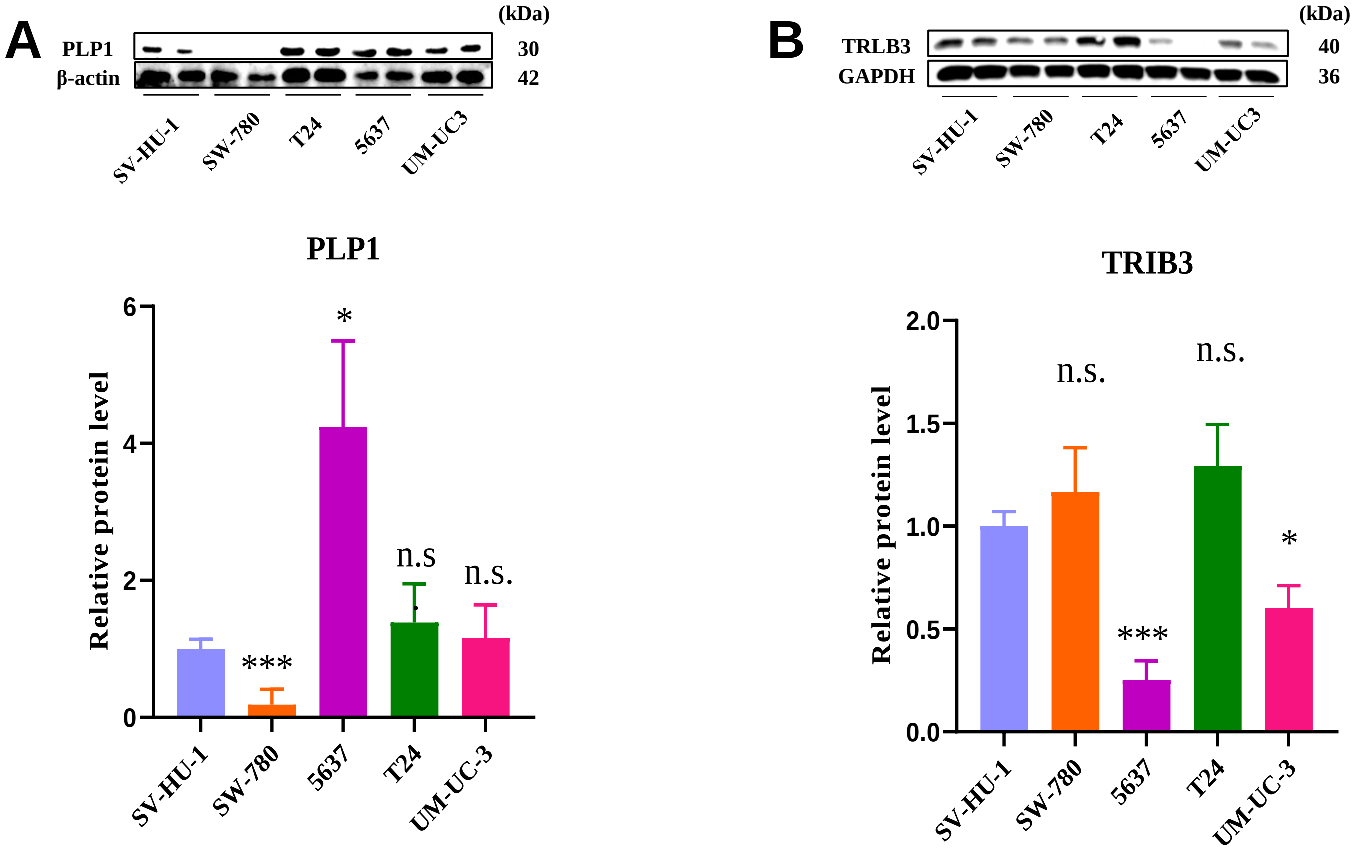

Figure S10 Protein expression and quantification of PLP1(A) and TRIB3 (B) in different bladder cancer cell lines. An unpaired t-test was used. * p<0.05; ** p<0.01*** p<0.001; n.s. no significant difference. Original gels are presented in Supplementary Figure expanded view 2 and 3.

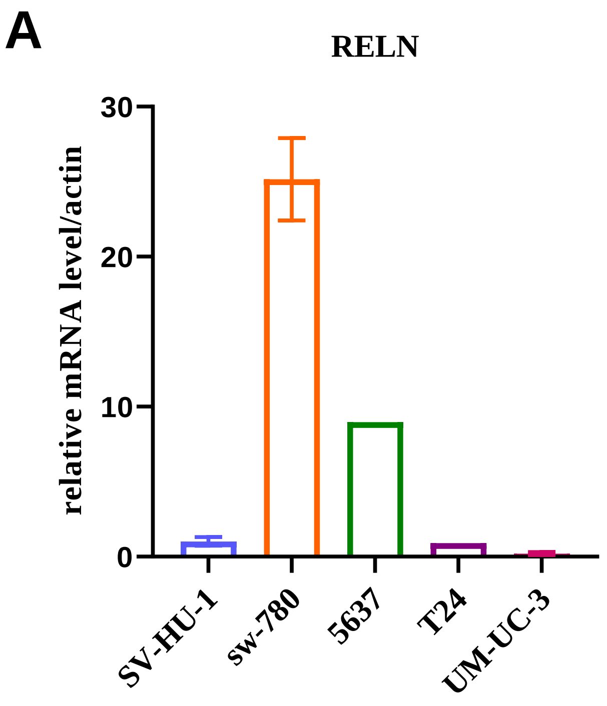

Figure S11: Transcription level of RELN (A) in different bladder cancer cell lines.
