# Supplementary material for: Identification and preliminary analysis of hub genes associated with bladder cancer progression by comprehensive bioinformatics analysis
Source: Sci Rep. 2024 Feb 2;14:2782. doi: 10.1038/s41598-024-53265-z (PMC10837156; doi:10.1038/s41598-024-53265-z)
Supplement: Supplementary file 3 — Supplementary Table 1. [file 41598_2024_53265_MOESM3_ESM.docx]

**Supplemental online Table 1: Correlation analysis between PLP1 and RELN and immune cell infiltrations for bladder cancer.**

TAM, tumor-associated macrophage; Th, T helper cell; Tfh, Follicular helper T cell; Treg, regulatory T cell; Cor, R value of Spearman’s correlation; None, correlation without adjustment. Purity, correlation adjusted by purity.

* *P* < 0.05; ** *P* < 0.01; *** *P* < 0.001; **** *P* < 0.0001.

| **Description** | **Gene markers** | **PLP1** | | | | RELN | | | |
| --- | --- | --- | --- | --- | --- | --- | --- | --- | --- |
|  |  | **None** | | **Purity** | | **None** | | **Purity** | |
|  |  | **Cor** | **P** | **Cor** | **P** | **Cor** | **P** | **Cor** | **P** |
| CD8+  T cell | CD8A | 0.184 | **** | 0.018 | 0.736 | 0.33 | **** | 0.145 | * |
|  | CD8B | 0.141 | ** | 0.025 | 0.632 | 0.265 | **** | 0.132 | * |
| Tcell  (general) | CD3D | 0.163 | *** | -0.024 | 0.642 | 0.294 | **** | 0.086 | 0.098 |
|  | CD3E | 0.234 | **** | 0.035 | 0.509 | 0.377 | **** | 0.173 | *** |
|  | CD2 | 0.213 | **** | 0.014 | 0.785 | 0.372 | **** | 0.176 | *** |
| B cell | CD19 | 0.422 | **** | 0.31 | **** | 0.402 | **** | 0.258 | **** |
|  | CD79A | 0.406 | **** | 0.281 | **** | 0.418 | **** | 0.268 | **** |
| Monocyte | CD86 | -0.009 | 0.938 | 0.104 | 0.381 | -0.229 | 0.042 | -0.143 | 0.229 |
|  | CD115 | -0.061 | 0.596 | 0.036 | 0.761 | -0.392 | *** | -0.388 | *** |
| TAM | CCL2 | 0.084 | 0.463 | 0.164 | 0.165 | -0.102 | 0.369 | 0.007 | 0.951 |
|  | CD68 | -0.012 | 0.914 | -0.102 | 0.389 | -0.431 | **** | -0.398 | *** |
|  | IL10 | 0.13 | 0.252 | 0.231 | 0.049 | 0.2 | 0.078 | -0.142 | 0.229 |
| M1 Macrophage | INOS | 0.021 | 0.855 | 0.049 | 0.678 | -0.086 | 0.449 | -0.087 | 0.465 |
|  | IRF5 | -0.13 | 0.254 | -0.077 | 0.519 | -0.176 | 0.122 | -0.122 | 0.302 |
|  | COX2 | 0.048 | 0.672 | 0.086 | 0.472 | 0.098 | 0.392 | 0.155 | 0.191 |
| M2 Macrophage | CD163 | 0.36 | **** | 0.197 | *** | 0.461 | **** | 0.266 | **** |
|  | VSIG4 | 0.349 | **** | 0.177 | *** | 0.439 | **** | 0.235 | **** |
|  | MS4A4A | 0.373 | **** | 0.213 | **** | 0.482 | **** | 0.298 | **** |
|  | CD66b | 0.024 | 0.635 | 0.039 | 0.450 | 0.085 | 0.087 | 0.118 | * |
| Neutrophils | CD11b | 0.313 | **** | 0.118 | * | 0.447 | **** | 0.`247 | **** |
|  | CCR7 | 0.216 | **** | 0.143 | ** | 0.106 | * | 0.044 | 0.405 |
| Natural killer cell | KIR2DL1 | 0.018 | 0.071 | -0.097 | 0.063 | 0.189 | *** | 0.059 | 0.257 |
|  | KIR2DL3 | 0.069 | 0.163 | -0.065 | 0.21 | 0.185 | *** | 0.033 | 0.534 |
|  | KIR2DL4 | -0.05 | 0.305 | -0.204 | **** | 0.124 | * | -0.044 | 0.403 |
|  | KIR3DL1 | 0.06 | 0.227 | -0.038 | 0.464 | 0.178 | *** | 0.077 | 0.143 |
|  | KIR3DL2 | 0.078 | 0.115 | -0.036 | 0.497 | 0.164 | *** | 0.043 | 0.415 |
|  | KIR3DL3 | -0.082 | 0.099 | -0.126 | * | -0.002 | 0.972 | -0.054 | 0.302 |
|  | KIR2DS4 | 0.009 | 0.85 | -0.111 | * | 0.106 | * | -0.033 | 0.542 |
|  | HLA-DPB1 | 0.291 | **** | 0.109 | * | 0.405 | **** | 0.211 | **** |
| Dendritic cell | HLA-DQB1 | 0.201 | **** | 0.01 | 0.843 | 0.333 | **** | 0.133 | * |
|  | HLA-DRA | 0.235 | **** | 0.05 | 0.335 | 0.374 | **** | 0.185 | *** |
|  | HLA-DPA1 | 0.246 | **** | 0.069 | 0.187 | 0.375 | **** | 0.193 | *** |
|  | BDCA-1 | 0.354 | **** | 0.254 | **** | 0.369 | **** | 0.26 | **** |
|  | BDCA-4 | 0.275 | **** | 0.194 | *** | 0.343 | **** | 0.239 | **** |
|  | CD11c | 0.297 | **** | 0.095 | 0.069 | 0.454 | **** | 0.238 | **** |
| Th1 | T-bet (TBX21) | 0.19 | *** | 0.006 | 0.914 | 0.347 | **** | 0.171 | *** |
|  | STAT4 | 0.182 | *** | -0.013 | 0.805 | 0.361 | **** | 0.17 | ** |
|  | STAT1 | 0.022 | 0.658 | -0.144 | ** | 0.169 | *** | -0.008 | 0.871 |
|  | IFN-γ (IFNG) | 0.008 | 0.866 | -0.157 | ** | 0.19 | *** | 0.011 | 0.827 |
|  | TNF-α (TNF) | -0.001 | 0.986 | 0.101 | * | 0.159 | ** | 0.029 | 0.583 |
| Th2 | GATA3 | -0.13 | ** | -0.33 | 0.529 | -0.240 | **** | -0.06 | 0.25 |
|  | STAT6 | -0.1 | * | -0.06 | 0.248 | -0.122 | * | -0.065 | 0.215 |
|  | STAT5A | 0.215 | **** | 0.107 | * | 0.259 | **** | 0.145 | ** |
|  | IL13 | 0.244 | **** | 0.152 | ** | 0.265 | **** | 0.174 | *** |
| Tfh | BCL6 | -0.017 | 0.734 | 0.005 | 0.931 | -0.034 | 0.493 | 0.009 | 0.857 |
|  | IL21 | 0.141 | ** | 0.06 | 0.249 | 0.171 | *** | 0.097 | 0.062 |
| Th17 | STAT3 | 0.148 | ** | 0.043 | 0.406 | 0.23 | **** | 0.117 | * |
|  | IL17A | -0.174 | *** | -0.235 | **** | -0.055 | 0.263 | -0.119 | * |
| Treg | FOXP3 | 0.26 | **** | 0.119 | * | 0.419 | **** | 0.282 | **** |
|  | CCR8 | 0.288 | **** | 0.167 | ** | 0.394 | **** | 0.259 | **** |
|  | STAT5B | 0.323 | **** | 0.326 | **** | 0.23 | **** | 0.268 | **** |
|  | TGFβ (TGFB1) | 0.069 | 0.162 | -0.01 | 0.846 | 0.118 | * | 0.015 | 0.777 |
| T cell exhaustion | PD-1 (PDCD1) | 0.165 | **** | -0.025 | 0.628 | 0.333 | **** | 0.134 | ** |
|  | CTLA4 | 0.186 | *** | 0.003 | 0.95 | 0.337 | **** | 0.151 | ** |
|  | LAG3 | 0.13 | ** | -0.049 | 0.349 | 0.287 | **** | 0.086 | 0.1 |
|  | TIM-3 (HAVCR2) | 0.252 | **** | 0.039 | 0.457 | 0.418 | **** | 0.202 | **** |
|  | GZMB | 0.112 | * | -0.103 | * | 0.267 | **** | 0.029 | 0.581 |

TAM, tumor-associated macrophage; Th, T helper cell; Tfh, Follicular helper T cell; Treg, regulatory T cell; Cor, R value of Spearman’s correlation; None, correlation without adjustment. Purity, correlation adjusted by purity.

* *P* < 0.05; ** *P* < 0.01; *** *P* < 0.001; **** *P* < 0.0001.
