# Supplementary material for: Identification and preliminary analysis of hub genes associated with bladder cancer progression by comprehensive bioinformatics analysis
Source: Sci Rep. 2024 Feb 2;14:2782. doi: 10.1038/s41598-024-53265-z (PMC10837156; doi:10.1038/s41598-024-53265-z)
Supplement: Supplementary file 4 — Supplementary Table 2. [file 41598_2024_53265_MOESM4_ESM.docx]

**Supplemental online Table 2: Correlation analysis between CDH19 and TRIB3 and immune cell infiltrations for bladder cancer.**

TAM, tumor-associated macrophage; Th, T helper cell; Tfh, Follicular helper T cell; Treg, regulatory T cell; Cor, R value of Spearman’s correlation; None, correlation without adjustment. Purity, correlation adjusted by purity.

* *P* < 0.05; ** *P* < 0.01; *** *P* < 0.001; **** *P* < 0.0001.

| **Description** | **Gene markers** | **CDH19** | | | | TRIB3 | | | |
| --- | --- | --- | --- | --- | --- | --- | --- | --- | --- |
|  |  | **None** | | **Purity** | | **None** | | **Purity** | |
|  |  | **Cor** | **P** | **Cor** | **P** | **Cor** | **P** | **Cor** | **P** |
| CD8+ T cell | CD8A | 0.171 | *** | 0.035 | 0.509 | 0.175 | *** | 0.09 | 0.084 |
|  | CD8B | 0.169 | *** | 0.083 | 0.111 | 0.091 | 0.065 | 0.025 | 0.639 |
| Tcell (general) | CD3D | 0.118 | * | -0.024 | 0.629 | 0.07 | 0.157 | -0.063 | 0.224 |
|  | CD3E | 0.189 | *** | 0.035 | 0.053 | 0.101 | * | -0.031 | 0.555 |
|  | CD2 | 0.164 | *** | 0.01 | 0.853 | 0.116 | * | -0.009 | 0.868 |
| B cell | CD19 | 0.338 | **** | 0.271 | **** | 0.133 | ** | 0.033 | 0.522 |
|  | CD79A | 0.295 | **** | 0.199 | *** | 0.092 | 0.062 | -0.032 | 0.543 |
| Monocyte | CD86 | 0.24 | **** | 0.097 | 0.063 | 0.201 | **** | 0.105 | 0.044 |
|  | CD115 | 0.106 | 0.351 | -0.063 | 0.599 | 0.033 | 0.77 | 0.096 | 0.419 |
| TAM | CCL2 | 0.265 | 0.018 | 0.15 | 0.206 | 0.257 | * | 0.331 | ** |
|  | CD68 | 0.212 | 0.061 | 0.125 | 0.291 | 0.046 | 0.686 | 0.102 | 0.391 |
|  | IL10 | 0.18 | 0.113 | 0.067 | 0.574 | 0.228 | ** | 0.317 | ** |
| M1 Macrophage | INOS | 0.054 | 0.637 | 0.081 | 0.493 | -0.059 | 0.604 | -0.041 | 0.728 |
|  | IRF5 | -0.012 | 0.916 | -0.111 | 0.352 | 0.132 | 0.244 | 0.189 | 0.11 |
|  | COX2 | 0.301 | ** | 0.283 | 0.015 | 0.35 | ** | 0.32 | ** |
| M2 Macrophage | CD163 | 0.289 | **** | 0.161 | *** | 0.297 | **** | 0.225 | **** |
|  | VSIG4 | 0.275 | **** | 0.141 | ** | 0.249 | **** | 0.161 | ** |
|  | MS4A4A | 0.292 | **** | 0.163 | ** | 0.272 | **** | 0.179 | *** |
|  | CD66b | 0.149 | ** | 0.178 | *** | 0.026 | 0.597 | 0.031 | 0.553 |
| Neutrophils | CD11b | 0.263 | **** | 0.135 | ** | 0.238 | **** | 0.145 | ** |
|  | CCR7 | 0.12 | * | 0.095 | 0.069 | -0.018 | 0.713 | -0.07 | 0.183 |
| Natural killer cell | KIR2DL1 | 0.038 | 0.442 | -0.059 | 0.257 | 0.091 | 0.069 | 0.039 | 0.453 |
|  | KIR2DL3 | 0.059 | 0.236 | -0.052 | 0.323 | 0.141 | ** | 0.075 | 0.151 |
|  | KIR2DL4 | -0.001 | 0.983 | -0.12 | * | 0.139 | ** | 0.083 | 0.112 |
|  | KIR3DL1 | 0.044 | 0.376 | -0.03 | 0.563 | 0.067 | 0.174 | 0.014 | 0.787 |
|  | KIR3DL2 | 0.043 | 0.389 | -0.035 | 0.499 | 0.082 | 0.092 | 0.023 | 0.656 |
|  | KIR3DL3 | -0.08 | 0.106 | -0.108 | * | 0.006 | 0.905 | -0.01 | 0.846 |
|  | KIR2DS4 | 0.042 | 0.4 | -0.041 | 0.432 | 0.143 | ** | 0.109 | * |
|  | HLA-DPB1 | 0.271 | **** | 0.06 | 0.25 | 0.197 | **** | 0.094 | 0.079 |
| Dendritic cell | HLA-DQB1 | 0.165 | *** | 0.013 | 0.801 | 0.218 | **** | 0.146 | ** |
|  | HLA-DRA | 0.181 | *** | 0.032 | 0.545 | 0.199 | **** | 0.101 | 0.052 |
|  | HLA-DPA1 | 0.184 | *** | 0.031 | 0.556 | 0.221 | **** | 0.134 | * |
|  | BDCA-1 | 0.216 | **** | 0.122 | * | -0077 | 0.12 | -0.164 | ** |
|  | BDCA-4 | 0.209 | **** | 0.13 | * | 0.241 | **** | 0.198 | *** |
|  | CD11c | 0.262 | **** | 0.114 | * | 0.263 | **** | 0.173 | *** |
| Th1 | T-bet (TBX21) | 0.165 | *** | 0.028 | 0.595 | 0.163 | *** | 0.056 | 0.282 |
|  | STAT4 | 0.206 | **** | 0.072 | 0.168 | 0.112 | * | 0.001 | 0.992 |
|  | STAT1 | 0.126 | * | 0.01 | 0.844 | 0.234 | **** | 0.173 | *** |
|  | IFN-γ (IFNG) | 0.022 | 0.652 | -0.094 | 0.071 | 0.173 | *** | 0.106 | * |
|  | TNF-α (TNF) | 0.135 | ** | 0.075 | 0.149 | 0.298 | **** | 0.239 | **** |
| Th2 | GATA3 | -0.12 | * | -0.05 | 0.339 | -0.224 | **** | -0.168 | ** |
|  | STAT6 | 0.049 | 0.321 | 0.13 | * | -0.238 | **** | -0.224 | **** |
|  | STAT5A | 0.226 | **** | 0.167 | ** | 0.039 | 0.43 | -0.051 | 0.329 |
|  | IL13 | 0.125 | * | 0.059 | 0.259 | -0.028 | 0.575 | -0.088 | * |
| Tfh | BCL6 | 0.004 | 0.93 | 0.013 | 0.802 | -0.208 | **** | -0.182 | *** |
|  | IL21 | 0.117 | * | 0.068 | 0.195 | 0.149 | ** | 0.13 | * |
| Th17 | STAT3 | 0.237 | **** | 0.188 | *** | 0.16 | ** | 0.089 | 0.087 |
|  | IL17A | -0.153 | ** | -0.161 | ** | -0.026 | 0.598 | -0.044 | 0.404 |
| Treg | FOXP3 | 0.24 | **** | 0.146 | ** | 0.132 | **** | 0.041 | 0.438 |
|  | CCR8 | 0.276 | **** | 0.194 | *** | 0.119 | * | 0.027 | 0.599 |
|  | STAT5B | 0.295 | **** | 0.303 | **** | 0.014 | 0.776 | 0.008 | 0.885 |
|  | TGFβ (TGFB1) | 0.008 | 0.879 | -0.068 | 0.191 | 0.107 | * | 0.053 | 0.313 |
| T cell exhaustion | PD-1 (PDCD1) | 0.139 | ** | -0.011 | 0.836 | 0.142 | ** | 0.027 | 0.611 |
|  | CTLA4 | 0.151 | ** | 0.013 | 0.807 | 0.134 | ** | 0.021 | 0.692 |
|  | LAG3 | 0.126 | ** | -0.02 | 0.699 | 0.256 | **** | 0.182 | *** |
|  | TIM-3 (HAVCR2) | 0.229 | **** | 0.067 | 0.198 | 0.245 | **** | 0.159 | ** |
|  | GZMB | 0.092 | 0.064 | -0.081 | 0.122 | 0.167 | *** | 0.076 | 0.146 |
